# Discovery, identification, and functional characterization of long noncoding RNAs in *Arachis hypogaea* L.

**DOI:** 10.1186/s12870-020-02510-4

**Published:** 2020-07-02

**Authors:** Haiying Tian, Feng Guo, Zhimeng Zhang, Hong Ding, Jingjing Meng, Xinguo Li, Zhenying Peng, Shubo Wan

**Affiliations:** 1 College of Life Science, Shandong University, Jinan, 250014 China; 2 Bio-Tech Research Center, Shandong Academy of Agricultural Science/Shandong Provincial Key Laboratory of Genetic Improvement, Ecology and Physiology of Crops, Jinan, 250014 China; 3 Peanut Research Institute of Shandong, Qingdao, 266100 China; 4 Shandong Academy of Agricultural Science, Jinan, 250014 China

**Keywords:** *Arachis hypogaea* L., Transcriptome analysis, lncRNAs, Alternative splicing, Organ-specific expression, Salt-stress

## Abstract

**Background:**

Long noncoding RNAs (lncRNAs), which are typically > 200 nt in length, are involved in numerous biological processes. Studies on lncRNAs in the cultivated peanut (*Arachis hypogaea* L.) largely remain unknown.

**Results:**

A genome-wide scan of the peanut (*Arachis hypogaea* L.) transcriptome identified 1442 lncRNAs, which were encoded by loci distributed over every chromosome. Long intergenic noncoding RNAs accounted for 85.58% of these lncRNAs. Additionally, 189 lncRNAs were differentially abundant in the root, leaf, or seed. Generally, lncRNAs showed lower expression levels, tighter tissue-specific expression, and less splicing than mRNAs. Approximately 44.17% of the lncRNAs with an exon/intron structure were alternatively spliced; this rate was slightly lower than the splicing rate of mRNA. Transcription at the start site event was the alternative splicing (AS) event with the highest frequency (28.05%) in peanut lncRNAs, whereas the occurrence rate (30.19%) of intron retention event was the highest in mRNAs. AS changed the target gene profiles of lncRNAs and increased the diversity and flexibility of lncRNAs, which may be important for lncRNAs to execute their functions. Additionally, a substantial number of the peanut AS isoforms generated from protein-encoding genes appeared to be noncoding because they were truncated transcripts; such isoforms can be legitimately regarded as a class of lncRNAs. The predicted target genes of the lncRNAs were involved in a wide range of biological processes. Furthermore, expression pattern of several selected lncRNAs and their target genes were examined under salt stress, results showed that all of them could respond to salt stress in different manners.

**Conclusions:**

This study provided a resource of candidate lncRNAs and expression patterns across tissues, and whether these lncRNAs are functional will be further investigated in our subsequent experiments.

## Background

The central dogma of molecular biology, which proposes the flow of information from DNA to RNA to protein [1], is no longer tenable with mounting number of RNAs not coding proteins. These noncoding RNAs (ncRNAs) have been classified in various ways in accordance with their locations, lengths, and biological functions [2]. Broadly, ncRNAs fall into two categories: house-keeping and regulatory [2, 3]. The latter can be divided into short ncRNAs (< 200 nt) and include short interfering RNAs (20–31 nt), small ncRNAs and long noncoding RNAs (> 200 nt, lncRNAs) [4]. Short interfering RNAs include small interfering RNAs, microRNAs (miRNAs), and PIWI-interacting RNAs; these sequences differ across the different eukaryotes where they are found [5]. LncRNAs are categorized into antisense lncRNAs, long intergenic ncRNAs (lincRNAs), and intronic lncRNAs (incRNAs) on the basis of their genomic origin and/or their orientation relative to their neighboring protein-encoding transcripts [3, 6–8].

Undoubtedly, advances in genomics and bioinformatics, particularly the extensive application of next-generation sequencing, have boosted the identification and annotation of ncRNAs that have been determined to participate in a range of regulatory roles rather than simply represent transcriptional noise [4–8]. Although lncRNAs might be the least well-studied of these ncRNAs, a growing body of evidence suggests that they exert their functions at the transcriptional, post-transcriptional, and epigenetic levels in fungi, plants, and animals [5, 9–15]. In particular, animal lncRNAs have been associated with aging, hematopoiesis, pri-miRNA processing, muscle differentiation, neural development, and immune responses [16–21]. Xist, one of the best-studied lncRNAs, silences transcription by directly interacting with SHARP, recruiting SMRT, activating HDAC3, and deacetylating histones to exclude Pol II across the X chromosome during development in female mammals [12]. While studies on plants are limited compared with those on humans and animals, tens of thousands of lncRNAs have been identified via RNA-seq and bioinformatics analyses in several plants, such as Arabidopsis [22], Medicago [23] soybean [9], rice [24], wheat [25], maize [26], tomato [27], mulberry [28], poplar [29], and sea buckthorn [11]. Furthermore, available researches have suggested that a small quantity of lncRNAs perform regulatory functions in plants similar to those in animals [30–32]. For example, the lncRNA *COOLAIR*, a long intronic noncoding RNA, is required for establishing stable repressive chromatin at FLC through its interaction with PRC2 [31]. In rice, Ding et al. found that the lncRNA long-day–specific male-fertility–associated RNA is essential for the normal pollen development of plants grown under long-day conditions [30]. And also, more and more researches suggest that lncRNAs play important roles in the regulation of gene expression in response to various stresses [33–38]. An Arabidopsis lncRNA, DROUGHT INDUCED lncRNA (DRIR), was a new positive regulator of the plant response to drought and salt stress [37]. DRIR expressed at a low level under control conditions but increased significantly under drought and salt stress as well as ABA treatment. And also, *drir*^*D*^ mutant or overexpressing DRIR in Arabidopsis could increase tolerance to drought and salt stress of the transgenic plants, RNA-seq results demonstrated that *DRIR* can modulate the expression of a series of genes involved in ABA signaling, water transport, and other stress-relief processes. Although a growing body of evidence supports the diverse potential roles of lncRNAs in plants, studies on lncRNAs in the cultivated peanut (*Arachis hypogaea* L.) largely remain unknown. Recently, Zhao et al. identified 50,873 lncRNAs of peanut from large-scale published RNA sequencing data, which belonged to 124 samples involving 15 different tissues, and predicted the co-expressions of targeted genes and 386 hub lncRNAs [39].

The peanut is an allotetraploid species derived from natural hybridization between the wild diploids *A. duranensis* and *A. ipaensis* [40]. The peanut ranks sixth among oilseed crops in terms of seed production; approximately two thirds of harvested peanut seed is used for oil production, with the residual seed cake used as a protein-rich meal for livestock [41, 42]. The genomic sequences of both of the progenitor species of the peanut have now been acquired [43], thus providing an opportunity to analyze the contribution of lncRNAs to the plant’s transcription and ultimately to its phenotype. Here, strand-specific sequence data obtained from the peanut were scanned for lncRNA content, and bioinformatics analysis combined with experiment were applied to illustrate the range of biological processes in which lncRNA activity is likely involved. Some new viewpoints were put forward.

## Results

### Peanut Transcriptome analysis

**Fig. 1:**
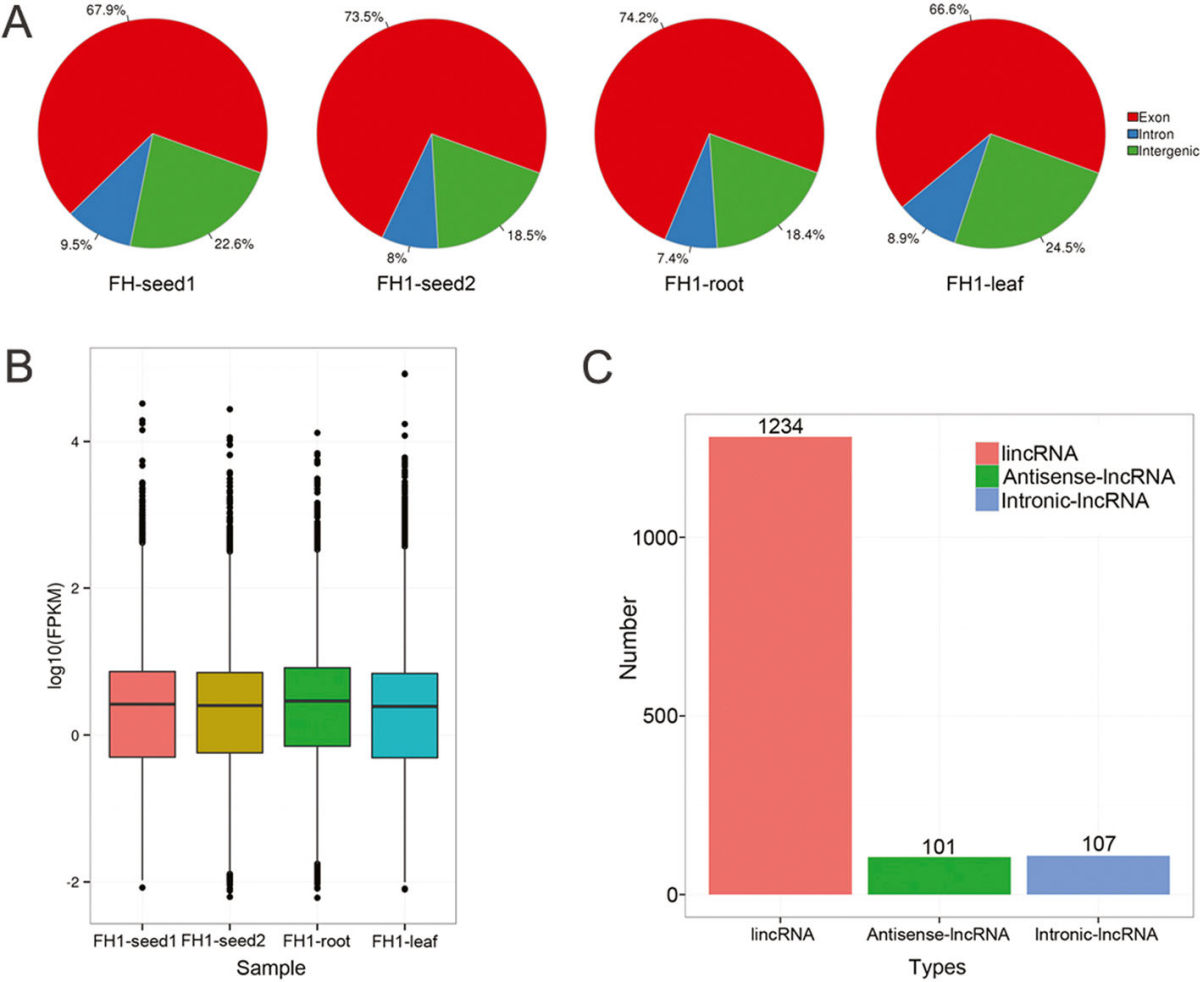
Long noncoding data compared with peanut reference genome sequence. **a** The distribution of mapped reads across the peanut reference genome; **b** FPKM_boxplots of each of the four libraries; **c** The abundance of each class of lncRNAs

After trimming adapter sequences and low-quality reads, over 11.14 Gb of clean data were acquired from each of the four libraries (FH1-seed1, FH1-seed2, FH1-root, and FH1-leaf) for further analysis. The reads were resolved into 203.8 billion paired-end reads with lengths of 125 bp. The individual libraries’ Q_30_ value ranged from 88.90 to 89.79%, and their GC content ranged from 44.48 to 50.48% (Additional file 1: Table S1). Between 79.91 and 84.16% of the reads were successfully aligned with the peanut reference genome sequence (Additional file 2: Table S2). In addition, the overall mapping results regarding the distribution of RNA-seq reads in the annotated protein coding genes (exonic and intronic) and intergenic regions should be presented in Fig. 1a. The majority of reads (66.60–74.25%) were mapped to the exonic sequence, with < 10% mapping to intronic sequence (Fig. 1a).

### Identification of LncRNAs

**Fig. 2:**
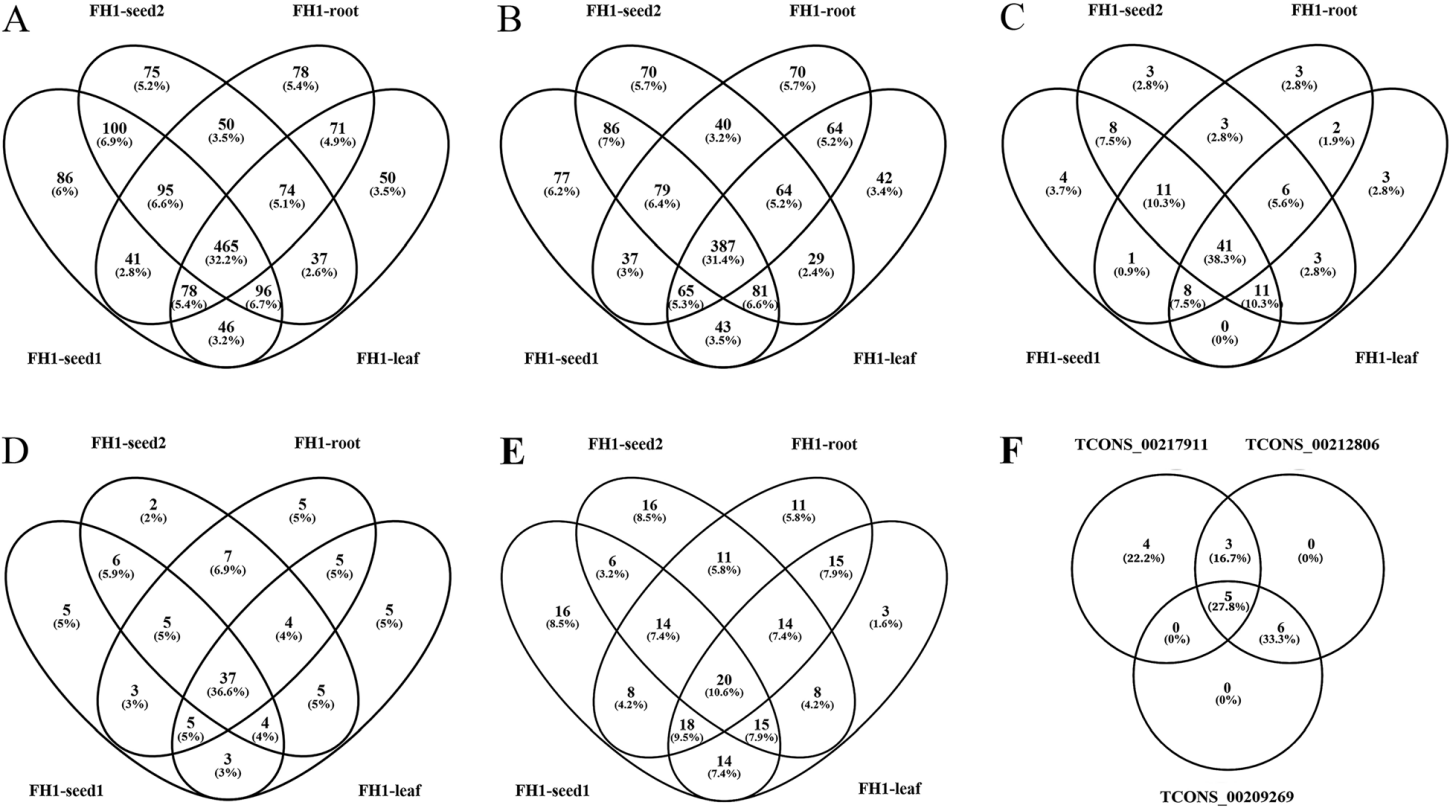
Overlap and uniqueness of the lncRNAs between organs. **a** The full set of lncRNAs; **b** lincRNAs; **c** intronicRNAs; **d** antisense-lncRNAs; **e** DALs

**Fig. 3:**
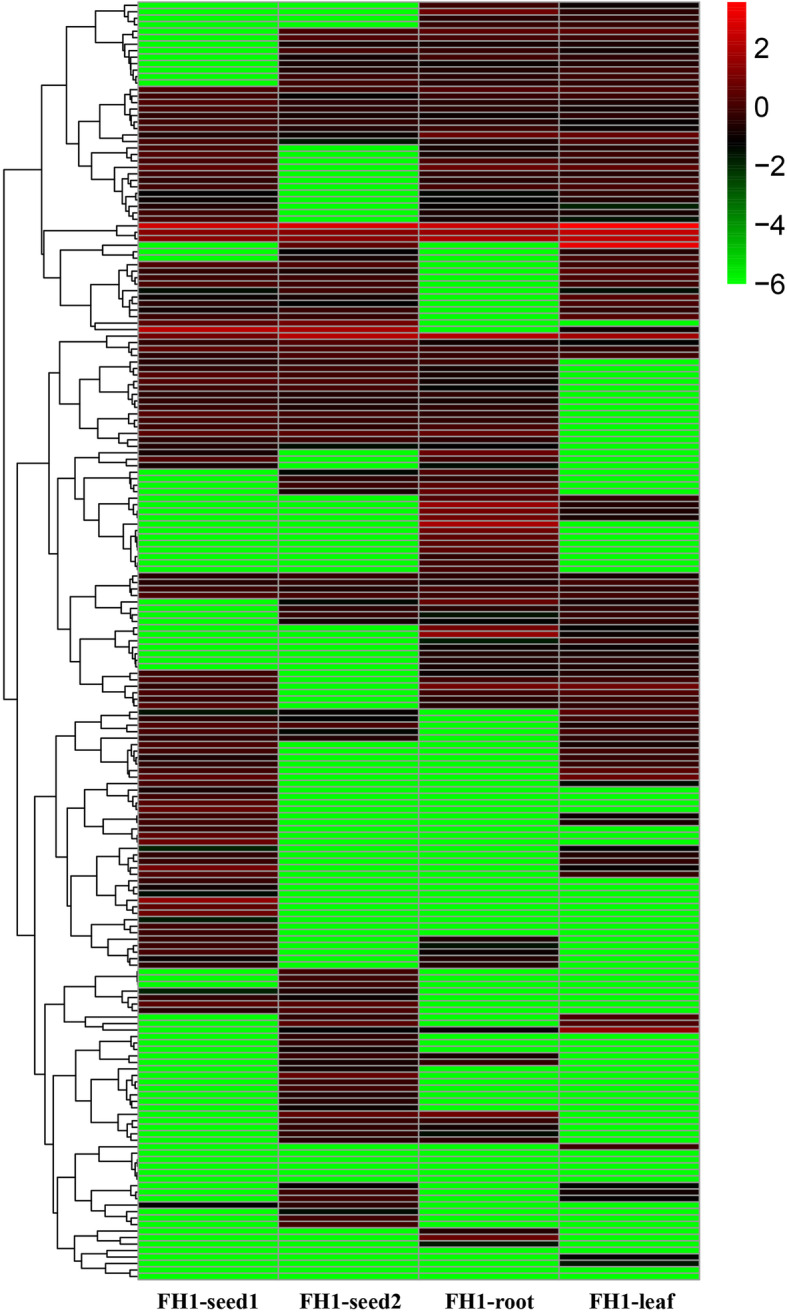
The abundance of the DALs across organs. The columns of the heat map represent each of the four libraries and the rows show the 189 DALs. The abundance of the DALs is indicated by the intensity of the color

A total of 1442 sequences remained after the imposition of the various criteria intended to identify putative lncRNAs (Additional file 3: Table S3); The FPKM values associated with each library suggested that the distribution of lncRNA abundance in the four libraries was broadly similar, although some evidence showed that the FH1-root library differed marginally with respect to their sequence distribution and their abundance (slightly higher) (Fig. 1b). Three types of lncRNA sequences were identified: lincRNAs, incRNAs, and antisense-lncRNAs, and lincRNAs accounted for 85.60% of the full set (Fig. 1c); of these sequences, 1007 were represented in FH1-seed1, 992 in FH1-seed2, 952 in FH1-root, and 917 in FH1-leaf (Fig. 2a). The number of sequences represented in all four libraries was 465, whereas the numbers of library-specific sequences were 86, 75, 78, and 50; 261 of the lncRNAs were specific to the developing seed (specific to either FH1-seed1 or FH1-seed2 or present in both libraries but absent from FH1-root and FH1-leaf). The number of lincRNAs represented in all four libraries was 387, whereas the numbers of library-specific lincRNAs were 77, 70, 70, and 42 (Fig. 2b). Substantial numbers of organ-specific intronic-RNAs and antisense-lncRNAs were also recognized (Fig. 2c, d). A total of 189 of the lncRNAs were classified as differentially abundant lncRNAs (DALs; Fig. 3; Additional file 4: Table S4). The number of DALs represented in all four libraries was 20, whereas the numbers of library-specific sequences were 16, 16, 11, and 3 (Fig. 2e).

### Genomic distribution of LncRNAs

The set of identified lncRNAs was transcribed from sequences distributed across all 20 chromosomes of the peanut genome, although a higher number was associated with the B subgenome than with the A subgenome chromosomes (863 vs 579, Additional file 5: Table S5). Chromosome Araip.B02 transcribed 112 of the identified lncRNAs against the 31 transcribed from chromosome Aradu.A07. The mean number of lncRNAs per hundred genes within the A subgenome was 2.57, whereas the equivalent statistic for the B subgenome was 3.37. The correlation between the number of lncRNAs and the number of transcribed genes (based on an FPKM threshold of 0.1) transcribed from each chromosome was + 0.61 (significant at *P* < 0.01).

### Comparison between mRNAs and LncRNAs

**Fig. 4:**
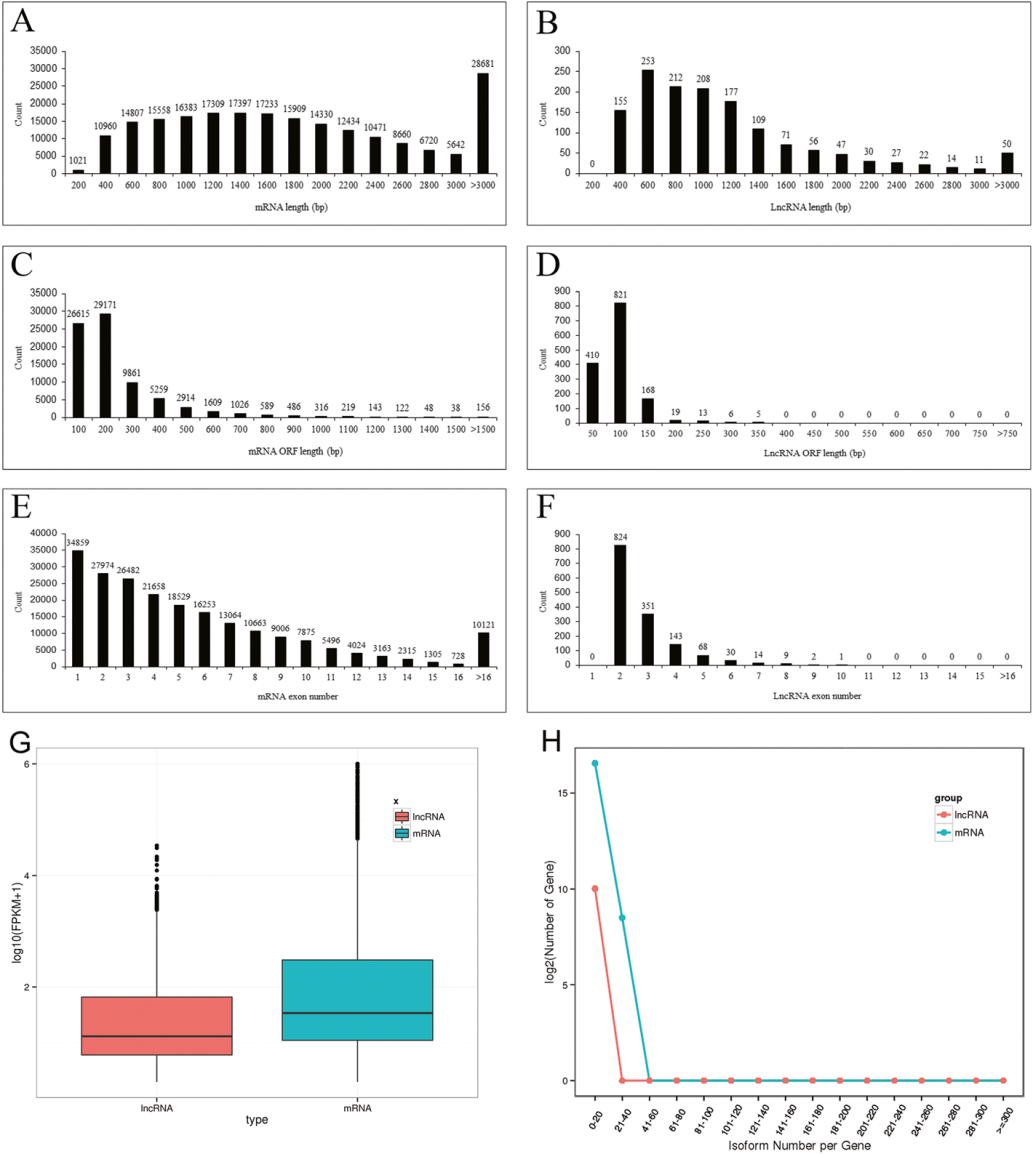
The population of peanut mRNAs and lncRNAs. **a** vs **b**, comparisons of transcript length; **c** vs **d**, ORF length; **e** vs **f**, exon number; **g**, transcript abundance; **h**, isoform density. The number on the bar (**a**-**f**) above the bar shows the transcript number

The sequences of 213,515 transcripts, generated from 55,621 genes, were acquired: this set of sequences, which included AS isoforms, is collectively referred here to “mRNAs”. The mean length of the mRNAs was 2017 nt, with the majority of their lengths falling in the range of 400–2600 nt (Fig. 4a), whereas the equivalent lengths of the lncRNAs were 1074 nt and 400–1400 nt. (Fig. 4b). The mean length of the open reading frames (ORFs) of the mRNAs was 240 nt, with the majority falling within the range of 100–300 nt, whereas that of the lncRNAs was 96 nt, with the length ranging from 50 nt to 150 nt (Fig. 4c, d). All of the lncRNAs, which included at least one intron, had fewer exons with a mean exon number of 2.77 than the mRNAs, which had a mean exon number of 5.48 (Fig. 4e, f). The abundance of the mRNAs was generally higher than that of the lncRNAs (Fig. 4g). The mRNAs generated a greater number of AS isoforms than did the lncRNAs (Fig. 4h).

### Predicting the target genes of the LncRNAs

**Fig. 5:**
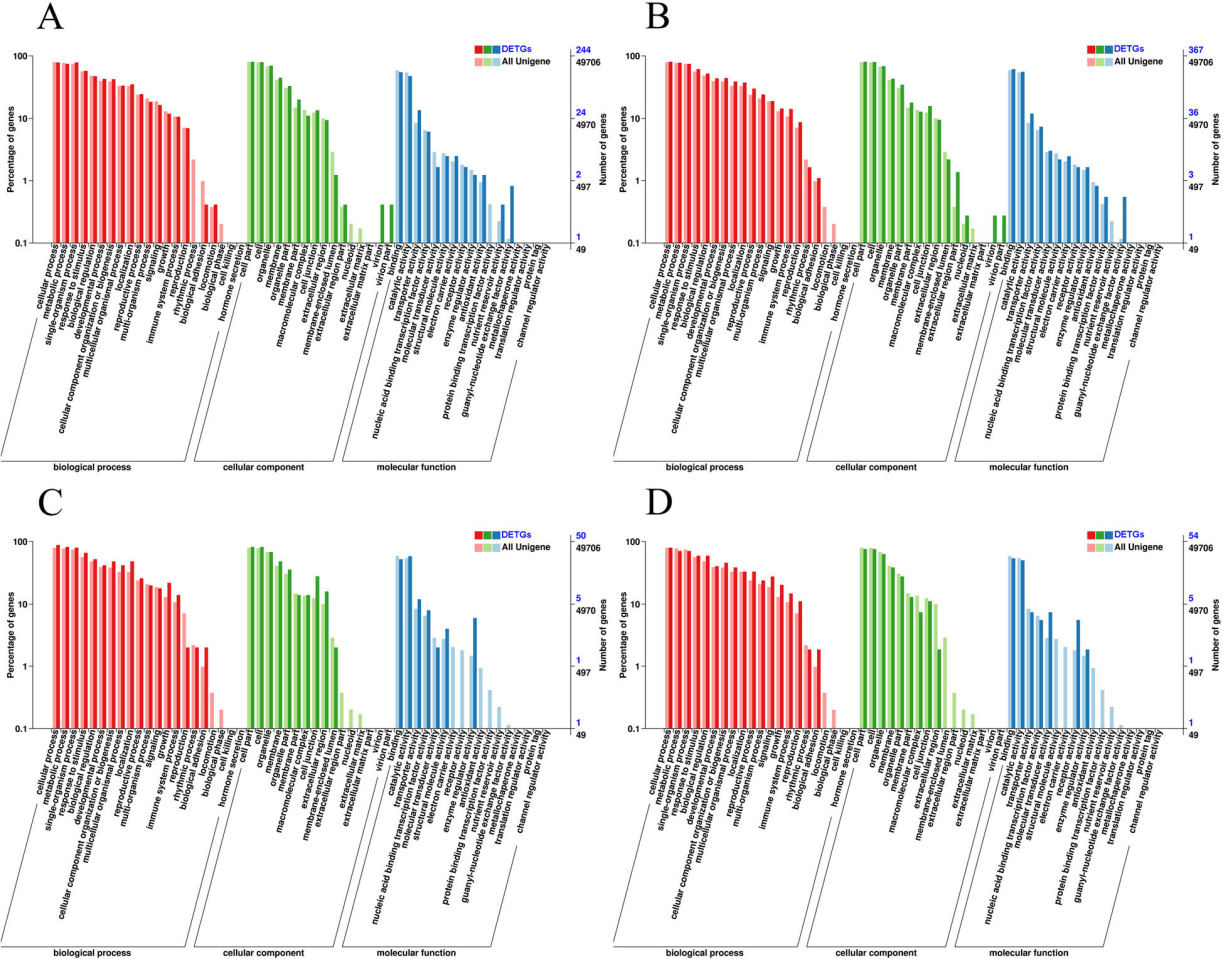
GO classification analysis of differentially expressed target genes of lncRNAs. **a**, FH1-seed1 and FH1-seed2; **b**, FH1-root and FH1-leaf; **c**, FH1-root and FH1-seed1&FH1-seed2; **d**, FH1-leaf and FH1-seed1&FH1-seed2. The ordinate is the enriched GO term and the abscissa is the number of differentially expressed target genes for the GO term. Different colors were used to distinguish biological processes (BPs), cellular components (CCs), and molecular functions (MFs)

To investigate the potential functions of the lncRNAs, their target genes were examined in *cis* (Additional file 6: Table S6) and in *trans* (Additional file 7: Table S7). All the target genes were annotated with their integrated function on the basis of five protein databases. A total of 11,765 target genes were obtained (Additional file 8: Table S8), with 9326 in FH1-seed1, 9415 in FH1-seed2, 8742 in FH1-root, and 8497 in FH1-leaf. Among these annotated target genes, 6550 were annotated in the COG database, 4209 in the GO database, 1922 in the KEGG database, 4422 in Swiss-Prot, and 10,019 in NR. Differential expressed target genes (DETGs) between these four libraries were analyzed, and the GO terms were used as an example to analyze the functional classification of the DETGs. In total, 49,706 unigenes were assigned GO terms, and the enriched terms differed in diverse tissues (Fig. 5). The number of DETGs enriched between FH1-seed1 and FH1-seed2 was 244 (Fig. 5a), with 367 between FH1-root and FH1-leaf (Fig. 5b), whereas the number of DETGs enriched between FH1-root and FH1-seed1&FH1-seed2, FH1-leaf and FH1-seed1&FH1-seed2 were 50 and 54, respectively (Fig. 5c, d). All the GO terms were classified into three major categories (biological process, cellular component, and molecular function), implied that lncRNAs might well be important for the regulation of a wide range of biological processes.

### Experimental validation of LncRNAs

In order to verify the reality of the predicted lncRNAs, seven of the lncRNAs (lncRNA1–7) were validated through PCR (Additional file 9: Table S9). The results of Sanger sequencing showed that five sequences identity with the relevant lncRNA sequence was very high (99.33–99.86%) except two (lncRNA1 and lncRNA2, Additional file 11: Fig. S1). When the PCR sequenced lncRNA1–7 were aligned to the corresponding genomic sequences of A and B sub-genomes, six of them (except lncRNA1) were well matched the predicted position on the corresponding chromosomes (Additional file 11: Fig. S1) LncRNA1 that shared only 85.68% identity with TCONS_00179561; a Blastn search of the genomic sequence located a highly homologous sequence (sequence identity: 99.6%) on Aradu.A03 in segment 130,713,152–130,713,435, whereas TCONS_00179561 was located in Araip.B03 131,673,533–131,674,406; thus lncRNA1 could not have been a product of TCONS_00179561, maybe PCR amplified its homologous genes. LncRNA2 shared 96.84% identity with TCONS_00109592. Blastn search located this sequence in the expected chromosome position (TCONS_00109592) but highlighted a 9 nt indel and several nucleotide polymorphisms between the genomic sequence and the lncRNA. We speculated that the product might be an AS isoform of lncRNA2. The differences between LncRNA3–7 and the corresponding genomic sequences were 1, 1, 2, 1, 6 nucleotides, respectively. For lncRNA4, the nucleotide is T at 609 nt of the cloned sequence as well as A01 subgenome and the nucleotide in RNA-seq was C. So, the discrepancy may well be due to the sequencing error in RNA-seq. For the others, the difference should be a result of sequencing error PCR amplification, because the sequences in RNA-seq were the same as their subgenomes (Fig. S1). Simply put, these differences maybe come from sequencing error, or PCR amplification. The successful validation of six out of the seven lncRNAs indicated that the prediction of the majority of the lncRNAs was credible.

### AS events of LncRNAs

Comparing the lncRNA and genomic DNA sequences suggested that AS is likely involved in their transcription. On the basis of the RNA-seq data, five AS events (transcription start site, TSS; transcription terminal site, TTS; exon skipping, ES; intron retention, IR; alternative exon, AE) were investigated (Table 1). Overall, 1811 AS events were involved in 637 lncRNAs, accounting for approximately 44.17% of the identified 1442 lncRNAs. Meanwhile, approximately 47.82% of mRNAs produced alternative transcripts; this rate is higher than that for lncRNAs. TSS was the AS event with the highest frequency (28.05%) in peanut lncRNAs, whereas the occurrence rate (30.19%) of IR was highest in mRNAs. Additionally, the frequency of the ES event was the lowest in lncRNAs and mRNAs.

**Table 1:** The comparison of AS events between lncRNAs and mRNAs

AS	lncRNA		mRNA	
Types	AS events	Percentage	AS events	Percentage
TSS	508	28.05%	16,942	19.87%
TTS	478	26.39%	13,914	16.32%
ES	39	2.15%	10,791	12.65%
IR	340	18.77%	25,748	30.19%
AE	446	24.63%	17,880	20.97%
Total	1811	100.00%	85,275	100.00%

**Fig. 6:**
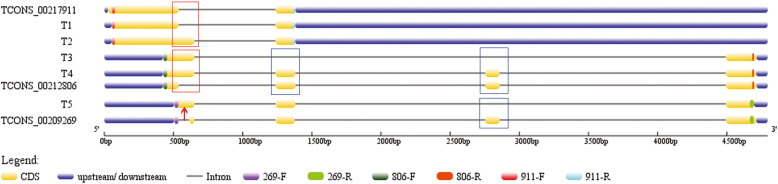
Identification of the AS isoforms of peanut lncRNAs. Gene structures of lncRNA AS isoforms. T1-T5: PCR products from Sanger sequencing; TCONS_00217911, TCONS_00209269 and TCONS_00212806 from the RNA-seq. Red rectangles indicate TSS type and blue ones show ES type. The red arrow shows IR type

Using the genomic segment 94,398,232–94,402,918 on chromosome Araip.B05 as an example, three lncRNAs (TCONS_00217911, TCONS_00212806, TCONS_00209269) (Additional file 3: Table S3) were speculated as a group of AS isoforms and verified by PCR test. Five transcripts, namely T1–T5, were sequenced (Fig. 6, Additional file 11: Fig. S2). T1 was identical to TCONS_00217911, T2 was an isoform of TCONS_00217911, and a TSS event was happened. T3 and T4 were AS isoforms of TCONS_00212806, and TSS and ES events were happened. T5 was an AS isoform of TCONS_00209269, and IR and ES events were happened. These alignments demonstrated that AS was a universal phenomenon in lncRNA, and may play important roles in their function regulation.

Another phenomenon was found related to the AS events of lncRNAs; that is, different AS isoforms of one lncRNA have different target genes. For example, the target genes of three lncRNAs (TCONS_00217911, TCONS_00212806, TCONS_00209269) were used for comparison. All of them have no *cis*-target genes (Additional file 6: Table S6) but have many *trans*-target genes (Additional file 7: Table S7). Each of them has 12, 14, and 11 *trans*-target genes, respectively. There were five *trans*-target genes belonged to three of them. Eight similar *trans*-target genes belonged to TCONS_00217911 and TCONS_00212806; five similar *trans*-target genes belonged to TCONS_00217911 and TCONS_00209269; 11 similar *trans*-target genes belonged to TCONS_00209269 and TCONS_00212806. Only TCONS_00217911 had four specific target genes (Fig. 2f). Other examples included TCONS_00011553, TCONS_00013006, and TCONS_00013007, which were all located on genome Aradu.A01 (99975619–99,981,506, Additional file 3: Table S3). The results of gene structure analysis showed that they had different AS isoforms (Additional file 12: Fig. S3A), and a total of 29 target genes were found (Additional file 6: Table S6, Additional file 7: Table S7). Among these target genes, 26 were represented in three of them, whereas only TCONS_00013006 had no specific target genes and the other two had only one (Additional file 12: Fig. S3B). AS can change the target-gene profiles of lncRNAs and increase the diversity and flexibility of lncRNA. This may be an important way for lncRNAs to execute their function.

### Protein-encoding genes as a source of LncRNAs

**Fig. 7:**
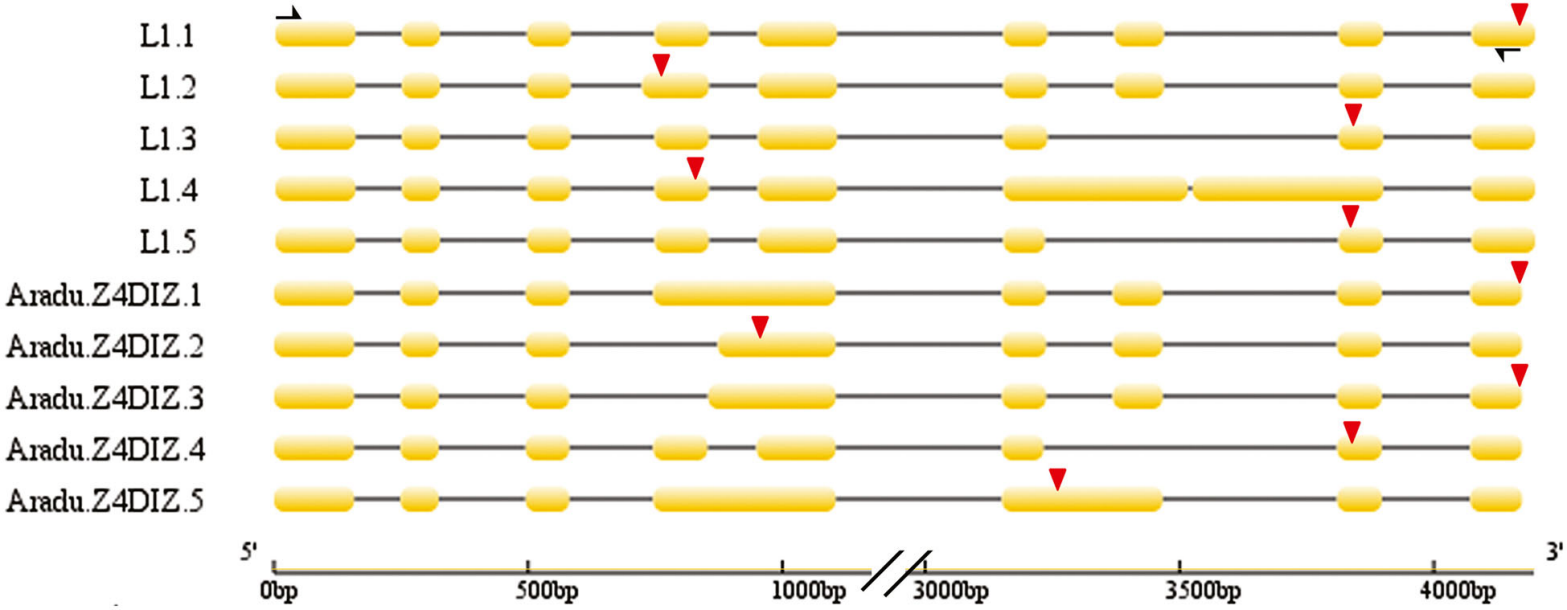
The gene structure of *Aradu.Z4DIZ* and its AS isoforms. L1.1-L1.5: PCR products from Sanger sequencing; Aradu.Z4DIZ.1–5: AS isoforms present in the transcriptomic sequence. Red triangle: stop codon. The black arrows show the location of the primers used for the PCR-based experimental validation

Not all AS isoforms encode a protein and are noncoding to a certain extent. A substantial number of the peanut AS isoforms generated from protein-encoding genes appeared to be noncoding because they were truncated transcripts; such isoforms can be legitimately regarded as a class of lncRNA. Several papers reported that approximately one-third of the alternative transcripts were likely noncoding [44, 45]. Here, a protein-encoding gene *Aradu.Z4DIZ,* which generates five transcripts, was selected as an example for validating this point (Fig. 7, Additional file 13: Fig. S4). Only two of the AS products (*Aradu.Z4DIZ.1* and *Aradu.Z4DIZ.3*) were predicted to encode complete proteins. The Sanger sequencing results of the amplified product generated from a primer pair directed at this gene revealed the presence of five isoforms: L1.5 matched *Aradu.Z4DIZ.4* given that both transcripts lacked the seventh exon. L1.1 encoded 232 amino acids (aa) and was 18 residues shorter than the predicted *Aradu.Z4DIZ.1* product. L1.2 encoded 118 aa, L1.4 encoded 177 aa, and L1.3 encoded 240 aa; the three encoded proteins were incomplete. The predicted product of *Aradu.Z4DIZ* is a diacylglycerol O-acyltransferase that carries a conserved LPLAT domain within its C-terminus; the truncated transcripts all lack the determinants of a complete LPLAT domain. This characteristic thereby compromises the functionality of their translation product.

### Validation of expression level of lncRNAs by qRT-PCR

**Fig. 8:**
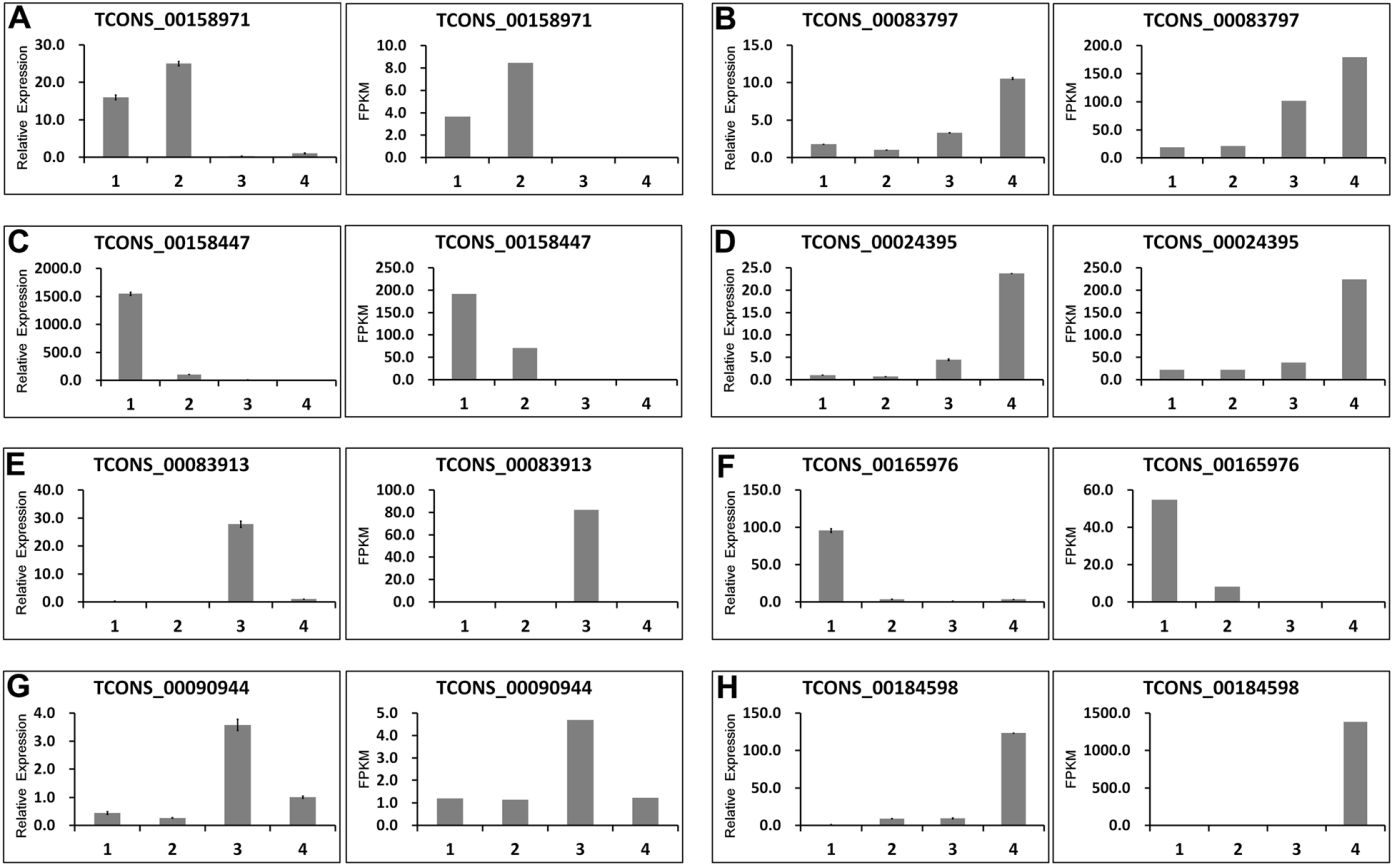
Validation of lncRNA expression using qRT-PCR. The abundance of each lncRNAs was deduced from RT-qPCR data (left-hand column) and from the RNA-seq data (right-hand column). The number 1, 2, 3, and 4 in the tissue label indicated FH1-root, FH1- leaf, FH1-seed1 nd FH1-seed2, respectively

To validate the abundance of lncRNAs, eight lncRNAs (lncRNA8–15) were randomly selected and analyzed through qRT-PCR (Additional file 9: Table S9). As shown in Fig. 8, the expression patterns of these lncRNAs were relatively consistent with RNA-seq results; this consistency indicated that the lncRNA expression patterns based on RNA-seq data are reliable.

### Coexpression of LncRNAs and their target genes by RT-PCR

**Fig. 9:**
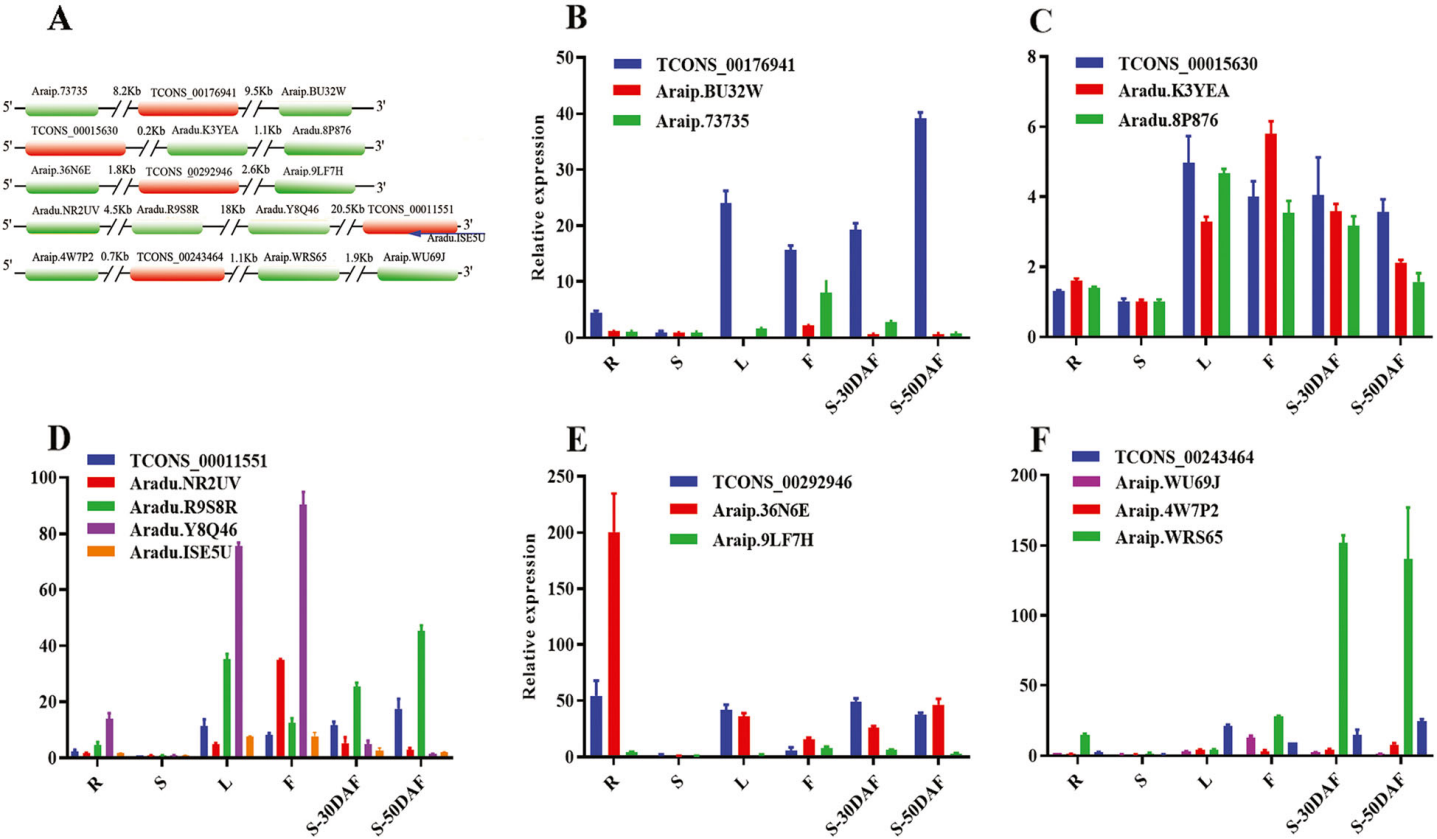
Comparison of the expression patterns of lncRNAs and the target genes by RT-PCR. **a**, The relative position of the five lncRNAs and their target genes. The blue arrow indicated *Aradu.ISE5U* which base-paired to the 3′-end of TCONS_00011551. **b**-**f**, Expression levels of five lncRNAs and their corresponding target genes in different tissues. R, S, L, F, S-30 DAF and S-50 DAF indicated root, stem, leaf, flower, seed from 30 DAF and 50 DAF, respectively

One lncRNA can interact with several gene targets and vice versa (Additional file 14: Fig. S5). Five lncRNAs (lncRNA16–20) and their target genes (Additional file 9: Table S9) were selected to verify the relationships between lncRNA and mRNA, the relative position of the five lncRNAs and their target genes are shown in Fig. 9a. TCONS_00176941 was located far from its two target genes at 8.2 and 9.5 Kb upstream and downstream, respectively. In all of the six organs, the expression level of TCONS_00176941 was higher than that of its two target genes (Fig. 9b). TCONS_00015630 was close to its two target genes downstream (Fig. 9a), and showed similar expression pattern with Aradu.8P876 in the six organs (Fig. 9c). TCONS_00011551 had three *cis-*target genes and one *trans*-target gene which showed different expression patterns in the six organs (Fig. 9a, d). TCONS_00292946 and its two target genes showed similar expression patterns in stems, leaves, flowers, and seeds at 30 day after flower (DAF) and in seeds at 50 DAF (Fig. 9e). TCONS_00243464 and its three target genes showed different expression patterns in six organs (Fig. 9f).

### Expression pattern analysis of LncRNAs and its target genes under salt stress

**Fig. 10:**
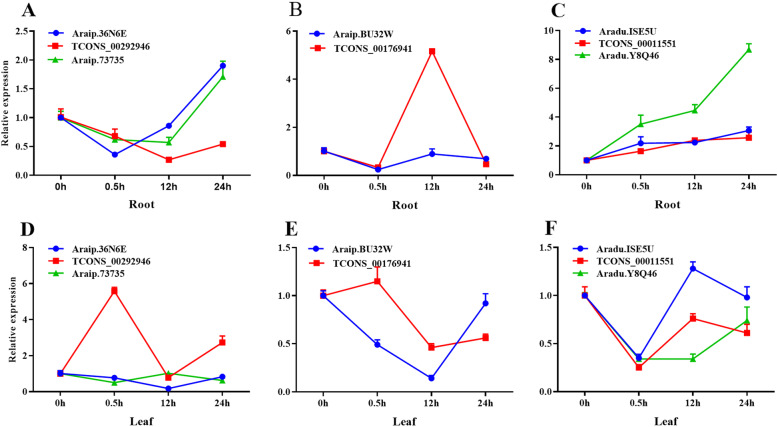
Expression patterns of three lncRNAs and their corresponding target genes under 1% NaCl stress inroot (**a**-**c**) and leaf (**d**-**f**), respectively. Data are presented as means ± SD of three independent replicates. *Ahactin* was used as the reference gene

LncRNAs play important role in stress resistance. Here, three lncRNAs and their target genes (Additional file 9: Table S9) were selected to test their expression patterns in different organs under salt stress using qRT-PCR (Fig. 10). These target genes were stress-related and with different expression level under salt stress, so the corresponding lncRNAs were selected for salt stress analysis. Results showed that these three lncRNAs and their target genes showed different expression patterns for salt treatment. In the roots, the expression level of TCONS_00292946 decreased within 12 h and then increased at 24 h (Fig. 10a). In the leaves, the expression level fluctuated (Fig. 10d). TCONS_00176941 showed opposing expression levels in the roots and leaves (Fig. 10b, e). The expression level of TCONS_00011551 slowly increased along with salt tress (Fig. 10c), but the expression level fluctuated in the leaves (Fig. 10f).

LncRNAs and their target genes showed different expression patterns in response to salt stress. TCONS_00292946 and its two target genes showed similar expression patterns in the roots (Fig. 10a) and opposing expression patterns in the leaves (Fig. 10d). TCONS_00176941 and Araip.BU32W showed similar expression patterns in the roots (Fig. 10b) and different expression patterns in the leaves (Fig. 10e). TCONS_00011551 and Aradu.ISE5U showed similar expression patterns in the roots and leaves, whereas TCONS_00011551 and Aradu.Y8Q46 showed different expression patterns in the leaves (Fig. 10c, f). The action mode of these lncRNAs in response to salt stress will be further studied.

## Discussion

Improvements in nucleotide sequencing technology have revealed the existence of lncRNAs, some of which act as the regulators of a range of eukaryotic cellular processes [15, 37, 46–48]. The number of lncRNAs identified by transcriptomic analyses reflects the depth of the sequencing method applied. A set of > 13,000 Arabidopsis lncRNAs [2], > 20,000 maize lncRNAs [26], and > 2000 rice lncRNAs has been reported [49]. Here, a survey of the peanut transcriptome revealed 1442 lncRNAs. By comparing our results with the published peanut lncRNAs of Zhao [39], in which 50,873 lncRNAs of peanut were identified from 124 samples involving 15 different tissues, we found 39 lncRNAs were identical, accounting for about 2.7% (data shown in Table S10). The low number of identified sequences may simply reflect the small scale of the experiment, which involved only four libraries as opposed to the 124 libraries underlying the above set of lncRNAs in Zhao’s [39]. In addition, the selection criteria applied here were more stringent than those commonly used. For example, by removing the restriction placed on the number of exons, > 10,000 lncRNAs would have been predicted (data not shown), among which a large number would have been false positives. When a small sample (seven sequences) of the set of lncRNAs was subjected to PCR-based validation, > 70% proved to be genuinely represented in the peanut transcriptome.

AS is adopted universally for post-transcriptional gene regulation [50, 51]. In plants, it is deployed to control aspects of growth, development, signal transduction, flowering, circadian clock function, and environmental cue responses [49, 52–55]. Similar to protein-encoding genes, the transcribed sequences for numerous lncRNAs developed AS variants in animals and plants [56–58]. For example, lncRNA-PXN-AS1, which lacks exon 4, binds to the coding sequences of PXN mRNA and inhibits PXN mRNA translation. By contrast, lncRNA-PXN-AS1, which contains exon 4, preferentially binds to the 3′ untranslated region of PXN mRNA, protects PXN mRNA from degradation, and thereby increases PXN expression [58]. Here, a number of AS isoforms were predicted from peanut RNA-seq data, and experiments via PCR validated these isoforms were real (Figs. 6, 7). Like AS of protein-coding genes, all kinds of AS types of protein-coding genes were found in lncRNAs (Table 1), but remarkable difference was also existed. We found that IR was the most occurred AS events in mRNA, with TSS the highest frequency in lncRNAs. AS events of lncRNAs can also increase the diversity of lncRNAs. It can change the target genes of a lncRNA (Fig. 2f, Additional file 12: Fig. S3), thus increase the flexibility of lncRNA. It may be an important regulation of lncRNAs.

Additionally, numerous protein-encoding genes transcribe diverse variants; several of these transcripts are unable to encode a functional protein because they represent the truncated forms of a fully functional mRNA (Fig. 7). These sequences could be regarded as another class of lncRNAs or pseudogene-derived lncRNAs in some sense [59]. Protein-coding mRNA transcripts can crosstalk with other mRNA transcripts by competing for common microRNAs [59]. The pseudogene PTENP1 has been hypothesized to be biologically active in prostate cancer cells by competitively binding to miR-17, miR-19, miR-21, and miR-26 families to regulate the cellular levels of PTEN and exert a growth-suppressive effect [60]. LncRNAs that were thought not to encode proteins could also translate small polypeptides to exert their function but not through the lncRNA itself [61, 62]. For example, Anderson et al. discovered a conserved micropeptide encoded by a skeletal muscle-specific RNA that was annotated as a putative lncRNA plays an important role in muscle performance [16]. Similarly, experimental evidence exists for the production of functional truncated polypeptides from AS variants derived from a protein-encoding gene. For example, the *A. salina CCA1* generates two isoforms, one of which is a full-size transcript (*CCA1α*), whereas the other is a truncated form (*CCA1β*) that lacks a functional N-terminal MYB DNA-binding domain [63]. The latter transcript inhibits CCA1α and LATE ELONGATED HYPOCOTYL transcription factors by forming nonfunctional heterodimers and is modulated by low temperatures. The human *USP2* gene generates seven AS variants, two of which are not translated into a functional protein [64]. The zebrafish gene *LGP2* forms three AS transcripts, of which only the full length version can confer protection against viral infection [65]. The distinction between coding and noncoding RNA is not as sharp as previously thought. Some protein-encoding genes generate noncoding AS variants, which can be recognized as lncRNAs; these sequences may be involved in gene regulation. With technological progress and unremitting effort, additional possible regulatory mechanisms of lncRNAs will be explicitly studied.

A growing body of research suggests that lncRNAs play important roles in plant stress resistance [33–36, 38, 47]. In this study, some peanut lncRNAs with the target genes were tested under salt stress, and the results showed that all of them changed their expression levels (Fig. 10). In poplar, more than 10,000 lncRNAs were identified, and approximately 40% of them responded to salt stress with tissue-specific expression patterns [34]. In cotton, lncRNA973 was localized in the nucleus and increased by salt treatment. The overexpression of lncRNA973 in Arabidopsis could increase salt tolerance, whereas the knockdown of lncRNA973 in cotton could reduce salt tolerance [36]. Furthermore, many studies reported that lncRNAs play important roles in plant defense against pathogens [66–68]. In tomato, a plausible model for TYLCV-induced diseases and host antiviral immunity was uncovered; that is, lncRNAs interact with the IR-derived vsRNAs to control disease development during TYLCV infection, which provide an effective strategy for the control of plant viral pathogens [67]. In rice, the connection between lncRNAs and the JA pathway in the regulation of bacterial blight was confirmed, which provided a novel insight into plant disease resistance [68]. lncRNAs have three salient features: low expression, lack of conservation between species, and tissue-specific expression patterns [23]. These features indicated that specific prevention and cure methods can be developed for a specific disease, according to specific species and lncRNAs. This will be a development direction for plant disease resistance research in the future.

## Conclusion

We identified 1442 lncRNAs in peanut transcriptome with strand-specific RNA-Seq technique. Among them, 189 lncRNAs were differentially abundant in the root, leaf, or seed. Approximately 44.17% of the lncRNAs with an exon/intron structure was alternatively spliced. This rate was slightly lower than the splicing rate of mRNA. AS changed the target-gene profiles of lncRNAs and increased the diversity and flexibility of lncRNAs. This may be an important way for lncRNAs to execute their functions. Additionally, protein-encoding genes produced many truncated transcripts by AS, which may be another source of lncRNAs. Some lncRNAs and their target genes were selected for salt response experiments. The results showed that all of them could respond to salt stress in different manners. Identification of peanut lncRNAs will have a strong impact on peanut development, growth, and stress resistance breeding.

## Materials and methods

### Materials

Peanut plants (cultivar ‘Fenghua-1’) were grown in a growth chamber with a photoperiod cycle of 16 h light at 26 °C and 8 h dark at 24 °C. Roots and leaves were collected from 12-day-old seedlings (names as FH1-root and FH1-leaf), and developing seeds were collected from plants at 30 days after flowering (DAF) (named as FH1-seed1) and 50 DAF (named as FH1-seed2).

### Library construction and sequencing

Total RNAs were isolated from FH1-root, FH1-leaf, FH1-seed1 and FH1-seed2 samples. Whole transcriptome library preparation and deep sequencing were performed as previously described [69]. Briefly, whole transcriptome libraries were prepared using NEBNext® Ultra™ Directional RNA Library Prep kit (New England Biolabs, Ipswich, MA, USA) following the manufacturer’s recommendations. Ultimately, sequencing was performed by imposing a paired-end 125 cycle rapid run on a HiSeq2500 platform (Illumina, San Diego, CA, USA). The raw data were deposited in the NCBI sequence read archive (http://www.ncbi.nlm.nih.gov/sra) with project PRJNA354652.

### Transcriptome assembly

Sequence data were stripped of adapter sequences and low-quality reads. The sequence data acquired from each library were aligned separately with peanut genome sequences [43] (https://www.peanutbase.org/) using TopHat2 software [70]. The aligned reads were assembled into a full transcriptome using the Cufflinks v2.2.1 program [71].

### Genome-wide identification of LncRNAs and alternative splicing events in peanut

The assembled transcripts were annotated using the Cuffcompare facility (http://cufflinks.cbcb.umd.edu/). Transcripts with lengths of more than 200 nt and at least two exons were selected as lncRNA candidates. Subsequently, the transcripts were analyzed using the coding potential calculator (score < 0) [72], the coding-noncoding index (score < 0) [73], the coding potential assessment tool [74], and Pfam (E-value < 0.001) [75] to remove all likely remaining protein-encoding genes. Only sequences that passed all of these four scans were considered as likely lncRNA candidates. Alternative splicing (AS) events were identified using ASTALAVISTA program (http://genome.crg.es/astalavista/). The diverse categories of AS events (TSS, TTS, ES, IR and AE) [76] were identified using a Perl script developed in house.

### Target gene prediction and functional annotation

Whether the lncRNAs acted in *cis* or in *trans* was predicted. The putative functions of the target genes were also predicted. Coding genes lying within 100 kb either at the 5′ upstream or 3′ downstream of each lncRNA were identified as potential *cis* targets [77] on the basis of whether the Pearson and Spearman correlation coefficients between the expression levels of these genes were ≥ 0.6 or ≤ − 0.6, and *P* < 0.05, whereas potential *trans* targets were predicted with Pearson correlation r > 0.9, P < 0.05; a gene coexpression network was constructed using the LncTar program [78], which searches for sequence complementarity between mRNAs and lncRNAs. Subsequently, the target genes were subjected to functional annotation analysis using the following databases: NCBI nonredundant protein sequences [79], KOG/COG [80], Swiss-Prot [81], KEGG [82], and GO [83]. The KOBAS software with default parameters was used to test the statistical enrichment of differentially expressed genes in KEGG pathways following the methods described by Mao et al. [84].

### Differential abundance of LncRNAs

Cufflinks v2.2.1 software was used to calculate FPKM values associated with lncRNAs and coding genes within each library. The analysis was performed using the EBseq (2010) R package [85]. *P*-values were adjusted to q-values [86]. Only the lncRNAs that met the criteria q-value < 0.01 and log2 (fold change) > 1 were considered as DALs.

### Validation of LncRNAs by PCR

Seven putative lncRNAs (lncRNA1–7) identified from RNA-seq data were validated using PCR assay (Additional file 9: Table S9). An aliquot of the total RNA used to construct the libraries was reverse-transcribed using a RevertAid First Strand cDNA Synthesis kit (Thermo Scientific™, Waltham, MA) following the manufacturer’s recommendations. Subsequently, the reaction products were diluted 20-fold to serve as the template in a PCR driven by the primer pairs given in Supplementary Table S9. Each 50 μL reaction comprised 5 μL of 10× TransTaq® HiFi Buffer II (Transgen Biotech, Beijing, China), 4 μL of 2.5 mΜ dNTP (each 10 μM), 1 μL of the forward primer and 1 μL of the reverse primer (each 10 μM), 4 μL of the cDNA template (50 ng/μL), 1 μL of TransTaq® HiFi DNA Polymerase (Transgen Biotech), and 35 μL of ddH_2_O. The amplified product was purified (TIANgel Midi Purification Kit DP209, TIANGEN Biotech, Beijing, China) and subcloned into the pEASY-T1 Cloning Vector (Transgen Biotech) for Sanger sequencing.

### Quantification of LncRNAs and target genes abundances using real-time quantitative PCR

The real-time quantitative PCR (RT-qPCR) platform was used to validate the abundance of a selection of the lncRNAs (lncRNA8–20, Supplementary Table S1) and adjacent target genes (*Araip.73735*, *Araip*.*BU32W*, *Aradu*.*K3YEA*, *Aradu*.*8P876*, *Araip*.*36N6E*, *Araip*.*9LF7H*, *Aradu*.*NR2UV*, *Aradu*.*R9S8R*, *Aradu*.*Y8Q46*, *Aradu*.*ISE5U*, *Araip*.*4W7P2*, *Araip*.*WRS65*, *Araip*.*WU69J*, Supplementary Table S9). We extracted total RNA of the tissues for qRT-PCR using TRIzol reagent (Invitrogen, Carlsbad, CA, USA) and the first-strand cDNA was synthesized by using MMLV reverse transcriptase with random hexamer primers according to the manufacturer’s protocol. All RT-qPCR reactions were conducted in triplicates for each cDNA sample using SYBR® Green Realtime PCR Master Mix (TOYOBO, Osaka, Japan) on ABI 7500 FAST real-time PCR platform. The specificity of PCR products was verified through melting curve analysis, and lncRNA and gene expression were quantified by using the 2^-ΔΔCt^ method, with the abundance of transcript of the gene *Actin-1* (XP_015966232.1) used for normalization. The primer sequences are shown in Supplementary Table S1.

### AS isoforms validation of LncRNAs by PCR

Three pair of specific primers (911-F/R, 269-F/R and 806-F/R, Additional file: Table S9) was designed to test for the presence of the AS isoforms of three lncRNA with the same genomic location (TCONS_00217911, Araip.B05 94,398,232–94,402,710; TCONS_00209269, Araip.B05 94,398,710–94,402,916; TCONS_00212806, Araip.B05 94,398,630–94,402,918). A reaction based on KOD-Plus-neo enzyme (TOYOBO, Osaka, Japan) was conducted according to manufacturers’ recommendations. After purification, the amplified product was transferred as above for sequencing.

### LncRNAs from protein-encoding genes

A pair of primers (Aradu.Z4DIZ-F/R, Additional file 9: Table S9) was designed to identify the AS isoforms generated from the gene *Aradu.Z4DIZ*. The PCR was based on the KOD-Plus-neo enzyme (TOYOBO, Osaka, Japan) with 50 μL reaction volume following manufacturers’ instructions. Amplicons were purified and transferred as above and sequenced.

### Salt treatment and validation by qRT-PCR

For salt treatment, peanut seedlings with a uniform growth status (2 weeks old, approximately 8 cm in height) were treated with 1% NaCl. Roots and leaves were harvested at 0, 0.5, 12, and 24 h after treatment. The harvested materials were snap-frozen in liquid nitrogen and stored at − 80 °C until required for RNA extraction. Total RNAs were prepared using a DP441 RNAprep Pure Plant kit (Tiangen, Beijing), and the resulting RNA converted into cDNA using a RevertAid First Strand cDNA Synthesis kit (K1621, Thermo Scientific™). RT-PCR analyses were conducted in triplicate using SYBR® Green Realtime PCR Master Mix (TOYOBO, Osaka, Japan) on ABI 7500 FAST real-time PCR platform.

Three groups of lncRNAs and their target genes (primers in Additional file 9: Table S9) were used for identification. Each 20 μL reaction contained 10 μL TaqMan Fast qPCR Master Mix, 0.4 μL of each non-labeled primer (10 μM each), 0.4 μL of fluorescently-labeled primer (10 μM), 2 μL cDNA (100 ng/μL) and 6.8 μL ddH_2_O. Relative transcript abundances were estimated using the 2^-ΔΔCT^ method [87]. Each reaction was run in triplicates for each cDNA sample.

## Supplementary information

**Additional file 1: Table S1.** Overview of the four total RNA-seq data sets.

**Additional file 2: Table S2.** RNA-seq data production and alignment results of four samples.

**Additional file 3: Table S3.** The detailed informations of 1442 lncRNAs.

**Additional file 4: Table S4.** 189 differentially expressed lncRNAs.

**Additional file 5: Table S5.** The distribution of lncRNAs in peanut chromosomes.

**Additional file 6: Table S6.***Cis*-target genes of lncRNAs.

**Additional file 7: Table S7.***Trans*-target genes of lncRNAs.

**Additional file 8: Table S8.** Integrated function annotation of the target genes.

**Additional file 9: Table S9.** Primers used in this paper.

**Additional file 10: Table S10.** Comparison with published peanut lncRNAs

**Additional file 11: Figure S1.** Sequence alignment of seven lncRNAs (lncRNA1–7) with their corresponding RNA-seq sequences.

**Additional file 12 Figure S2.** Sanger sequencing of five lncRNAs (T1–5).

**Additional file 13: Figure S3.** AS Events of LncRNAs and different AS isoforms had different target genes.

**Additional file 14: Figure S4.** Sanger sequencing of five lncRNAs (L1.1–1.5).

**Additional file 15: Figure S5.** The lncRNAs-protein interaction networks.

## Data Availability

Transcriptome raw data of *A. hypogaea* are available at NCBI project PRJNA354652 with accession number SRR5053815, SRR5054076, SRR5054058 and SRR5054059.

## Abbreviations

AE: Alternative exon ends
AS: Alternative splicing
DAF: day after flower
DETGs: Differential expressed target genes
ES: Exon skipping
FPKM: Fragments per kilobase of exon per million fragments mapped
Go: Gene Ontology
incRNAs: intronic lncRNAs
IR: Intron retention
KEGG: Sequences Kyoto Encyclopedia of Genes and Genomes
KOG/COG: Clusters of Orthologous Groups of proteins
lincRNAs: long intergenic ncRNAs
lncRNAs: long noncoding RNAs
miRNAs: microRNAs
ncRNAs: noncoding RNAs
Nr: non-redundant protein sequences
ORFs: Open reading frames
PCR: Polymerase Chain Reaction
RT–PCR: Reverse Transcription-Polymerase Chain Reaction
TSS: Transcription start site
TTS: Transcription terminal site
