# Supplementary material for: Discovery, identification, and functional characterization of long noncoding RNAs in Arachis hypogaea L
Source: BMC Plant Biol. 2020 Jul 2;20:308. doi: 10.1186/s12870-020-02510-4 (PMC7330965; doi:10.1186/s12870-020-02510-4)
Supplement: Supplementary file 11 — Additional file 11: Figure S1. Sequence alignment of seven lncRNAs (lncRNA1–7) with their corresponding RNA-seq sequences. [file 12870_2020_2510_MOESM11_ESM.pdf]

The images bellow shows alignments of lncRNA1-7 against RNA-seq and the corresponding genomic sequences.

Alignments of lncRNA1-7 against RNA-seq sequences.

lncRNA1 against TCONS_00179561

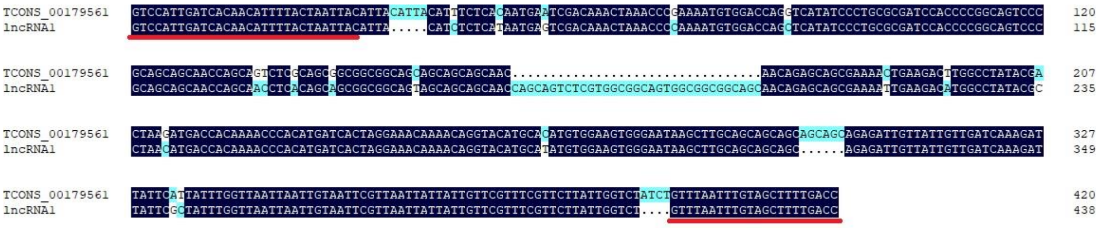

lncRNA2 against TCONS_00109592

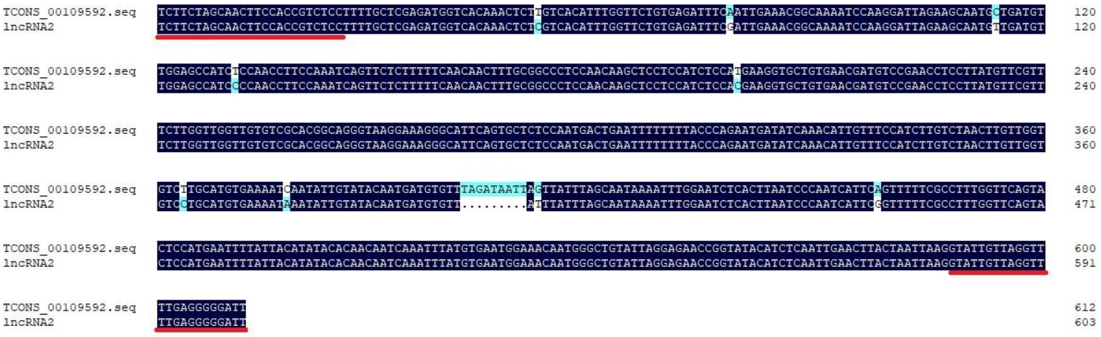

lncRNA3 against TCONS_00133884

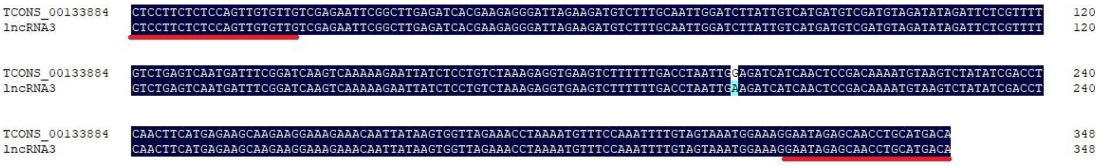

lncRNA4 against TCONS_00009270

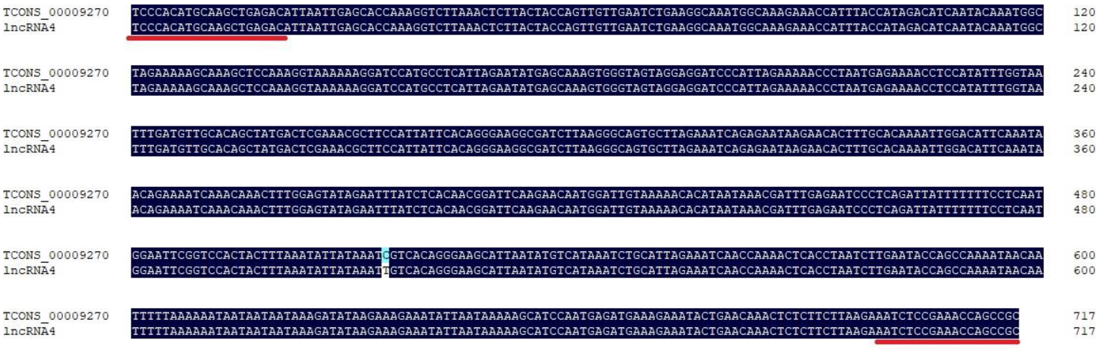

lncRNA5 against TCONS_00262811

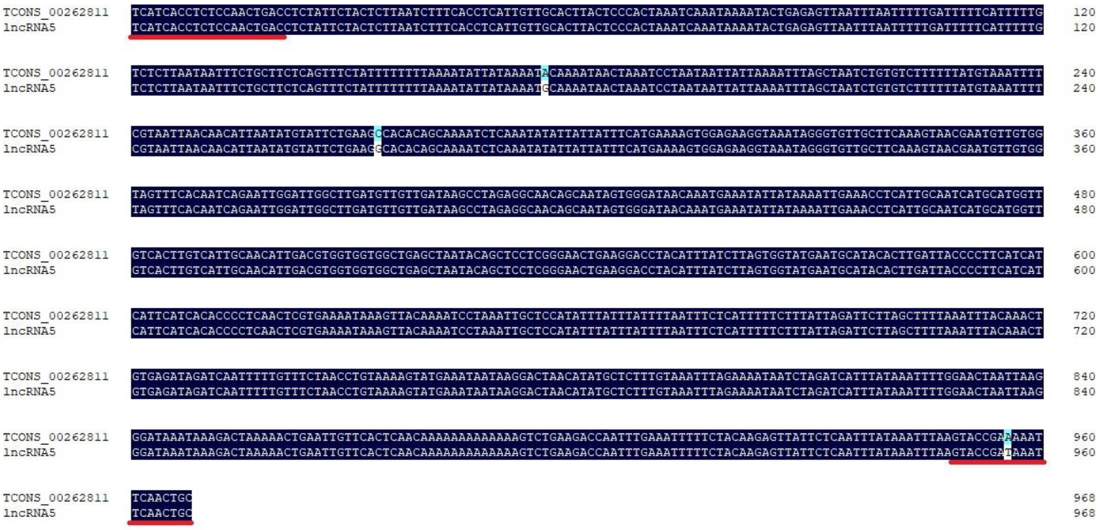

lncRNA6 against TCONS_00236479

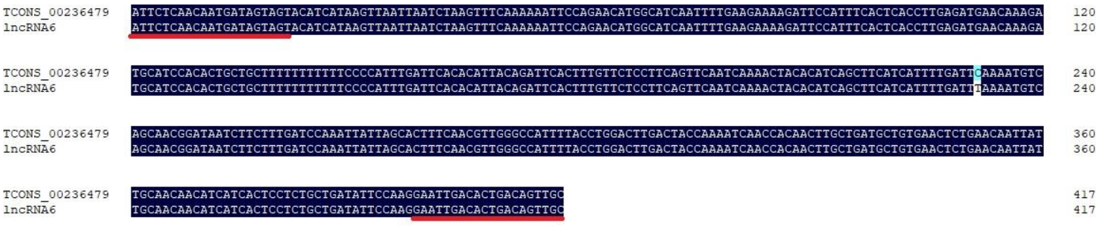

lncRNA7 against TCONS_00286692

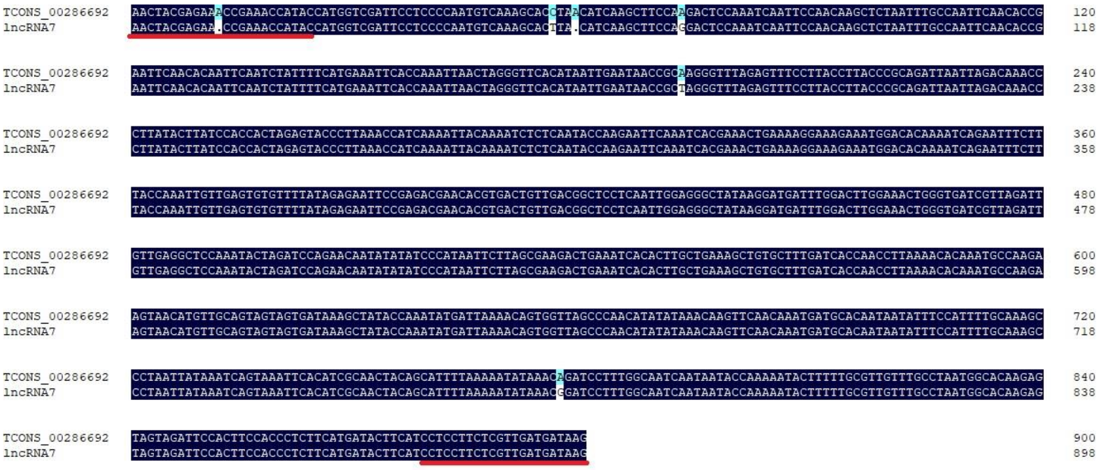

Alignments of lncRNA1-7 against the corresponding genomic sequences.

lncRNA1 against Aradu.A03: 130713152 – 130713435.

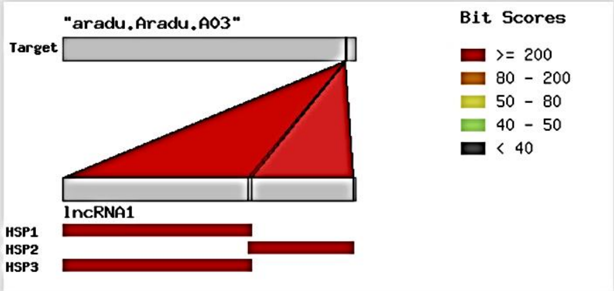

Alignment

HSP 1

Identity= 283/284 (99.65%) , Positive= 283/284 (99.65%) Query Matches 1 to 284 Hit Matches = 130713152 to 130713435

```
Query:          1 GTCCATTGATCACAACATTTTACTAATTACATTACATCTCTCATAATGAGTCGACAAACT 60
                  ||||||||||||||||||||||||||||||||||||||||||||||||||||||||||||
Sbjct:  130713152 GTCCATTGATCACAACATTTTACTAATTACATTACATCTCTCATAATGAGTCGACAAACT 130713211

Query:         61 AAACCCCAAAATGTGGACCAGCTCATATCCCTGCGCGATCCACCCCGGCAGTCCCGCAGC 120
                  ||||||||||||||||||||||||||||||||||| ||||||||||||||||||||||||
Sbjct:  130713212 AAACCCCAAAATGTGGACCAGCTCATATCCCTGCGTGATCCACCCCGGCAGTCCCGCAGC 130713271

Query:        121 AGCAACCAGCAACCTCACAGCAGCGGCGGCAGTAGCAGCAGCAACCAGCAGTCTCGTGGC 180
                  ||||||||||||||||||||||||||||||||||||||||||||||||||||||||||||
Sbjct:  130713272 AGCAACCAGCAACCTCACAGCAGCGGCGGCAGTAGCAGCAGCAACCAGCAGTCTCGTGGC 130713331

Query:        181 GGCAGTGGCGGCGGCAGCAACAGAGCAGCGAAAATTGAAGACATGGCCTATACGCCTAAC 240
                  ||||||||||||||||||||||||||||||||||||||||||||||||||||||||||||
Sbjct:  130713332 GGCAGTGGCGGCGGCAGCAACAGAGCAGCGAAAATTGAAGACATGGCCTATACGCCTAAC 130713391

Query:        241 ATGACCACAAAACCCACATGATCACTAGGAAACAAAACAGGTAC 284
                  ||||||||||||||||||||||||||||||||||||||||||||
Sbjct:  130713392 ATGACCACAAAACCCACATGATCACTAGGAAACAAAACAGGTAC 130713435
```

HSP 2

Identity= 156/158 (98.73%) , Positive= 156/158 (98.73%) Query Matches 279 to 436 Hit Matches = 130713529 to 130713686

```
Query:        279 AGGTACATGCATATGTGGAAGTGGGAATAAGCTTGCAGCAGCAGCAGAGATTGTTATTGT 338
                  ||||||||||||||||||||||||||||||||||||||||||||||||||||||||||||
Sbjct:  130713529 AGGTACATGCATATGTGGAAGTGGGAATAAGCTTGCAGCAGCAGCAGAGATTGTTATTGT 130713588

Query:        339 TGATCAAAGATTATTCGCTATTTGGTTAATTAATTGTAATTCGTTAATTATTATTGTTCG 398
                  ||||||||||||||||  ||||||||||||||||||||||||||||||||||||||||||
Sbjct:  130713589 TGATCAAAGATTATTCATTATTTGGTTAATTAATTGTAATTCGTTAATTATTATTGTTCG 130713648

Query:        399 TTTCGTTCTTATTGGTCTGTTTAATTTGTAGCTTTTGA 436
                  ||||||||||||||||||||||||||||||||||||||
Sbjct:  130713649 TTTCGTTCTTATTGGTCTGTTTAATTTGTAGCTTTTGA 130713686
```

HSP 3

Identity= 243/284 (85.56%) , Positive= 243/284 (85.56%) Query Matches 1 to 284 Hit Matches = 130744039 to 130744303

```
Query:          1 GTCCATTGATCACAACATTTTACTAATTACATTACATCTCTCATAATGAGTCGACAAACT 60
                  ||||||||||||||||||||||||||||||||||||||||||| ||||||||||||||||
Sbjct:  130744039 GTCCATTGATCACAACATTTTACTAATTACATTACATCTCTCACAATGAGTCGACAAACT 130744098

Query:         61 AAACCCCAAAATGTGGACCAGCTCATATCCCTGCGCGATCCACCCCGGCAGTCCCGCAGC 120
                  ||||||||||||||||| |||||||||||||| |||||||||||||||||||||||||||
Sbjct:  130744099 AAACCCCAAAATGTGGATCAGCTCATATCCCTTCGCGATCCACCCCGGCAGTCCCGCAGC 130744158

Query:        121 AGCAACCAGCAACCTCACAGCAGCGGCGGCAGTAGCAGCAGCAACCAGCAGTCTCGTGGC 180
                  |||||||||||  |||||||  |||||  ||||  ||||                  ||
Sbjct:  130744159 AGCAACCAGCAGTCTCACAGTGGCGGCAACAGTGACAGC------------------GGT 130744200

Query:        181 GGCAGTGGCGGCGGCAGCAACAGAGCAGCGAAAATTGAAGACATGGCCTATACGCCTAAC 240
                  ||||| | | ||  ||||  |||||||||||||| |||||||||||||||||||||||||
Sbjct:  130744201 GGCAGCGACAGCAACAGCGGCAGAGCAGCGAAAACTGAAGACATGGCCTATACGCCTAAC 130744260

Query:        241 ATGACCACAAAACCCACATGATCACTAGGAAACAAAACAGGTAC 284
                  |||||||||||||||| ||| ||||||||||||||||||| |||
Sbjct:  130744261 ATGACCACAAAACCCA-ATGGTCACTAGGAAACAAAACAGTTAC 130744303
```

lncRNA2 against Aradu.A08: 11755848-11759272

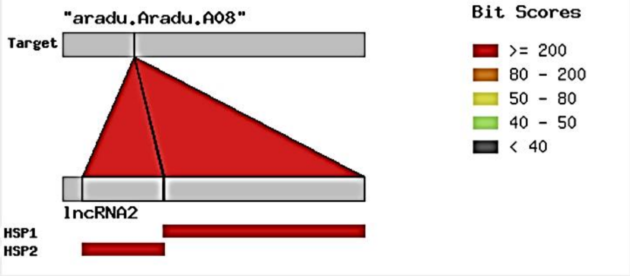

Alignment

HSP 1

Identity= 398/411 (96.84%) , Positive= 398/411 (96.84%) Query Matches 202 to 603 Hit Matches = 11758842 to 11759252

```
Query:         202 AGGTGCTGTGAACGATGTCCGAACCTCCTTATGTTCGTTTCTTGGTTGGTTGTGTCGCAC 261
                   ||||||||||||||||||||||||||||||||||||||||||||||||||||||||||||
Sbjct:    11758842 AGGTGCTGTGAACGATGTCCGAACCTCCTTATGTTCGTTTCTTGGTTGGTTGTGTCGCAC 11758901

Query:         262 GGCAGGGTAAGGAAAGGGCATTCAGTGCTCTCCAATGACTGAATTTTTTTTACCCAGAAT 321
                   ||||||||||||||||||||||||||||||||||||||||||||||||||||||||||||
Sbjct:    11758902 GGCAGGGTAAGGAAAGGGCATTCAGTGCTCTCCAATGACTGAATTTTTTTTACCCAGAAT 11758961

Query:         322 GATATCAAACATTGTTTCCATCTTGTCTAACTTGTTGGTGTCCTGCATGTGAAAATAAAT 381
                   |||||||||||||||||||||||||||||||||||||||||| ||||||||||||| |||
Sbjct:    11758962 GATATCAAACATTGTTTCCATCTTGTCTAACTTGTTGGTGTCTTGCATGTGAAAATCAAT 11759021

Query:         382 ATTGTATACAATGATGTG---------TTATTTATTTAGCAATAAAATTTGGAATCTCAC 432
                   ||||||||||||||||||         ||| |||||||||||||||||||||||||||||
Sbjct:    11759022 ATTGTATACAATGATGTGTTTAGATAATTAGTTATTTAGCAATAAAATTTGGAATCTCAC 11759081

Query:         433 TTAATCCCAATCATTCGGTTTTTCGCCTTTGGTTCAGTACTCCATGAATTTTATTACATA 492
                   |||||||||||||||| |||||||||||||||||||||||||||||||||||||||||||
Sbjct:    11759082 TTAATCCCAATCATTCAGTTTTTCGCCTTTGGTTCAGTACTCCATGAATTTTATTACATA 11759141

Query:         493 TACACAACAATCAAATTTATGTGAATGGAAACAATGGGCTGTATTAGGAGAACCGGTATA 552
                   ||||||||||||||||||||||||||||||||||||||||||||||||||||||||||||
Sbjct:    11759142 TACACAACAATCAAATTTATGTGAATGGAAACAATGGGCTGTATTAGGAGAACCGGTATA 11759201

Query:         553 CATCTCAATTGAACTTACTAATTAAGGTATTGTTAGGTTTTGAGGGGGATT 603
                   |||||||||||||||||||||||||||||||||||||||||||||||||||
Sbjct:    11759202 CATCTCAATTGAACTTACTAATTAAGGTATTGTTAGGTTTTGAGGGGGATT 11759252
```

HSP 2

Identity= 162/167 (97.01%) , Positive= 162/167 (97.01%) Query Matches 39 to 205 Hit Matches = 11756140 to 11756306

```
Query:          39 GGTCACAAACTCTCGTCACATTTGGTTCTGTGAGATTTCGATTGAAACGGCAAAATCCAA 98
                   ||||||||||||| ||||||||||||||||||||||||| ||||||||||||||||||||
Sbjct:    11756140 GGTCACAAACTCTTGTCACATTTGGTTCTGTGAGATTTCAATTGAAACGGCAAAATCCAA 11756199

Query:          99 GGATTAGAAGCAATGTTGATGTTGGAGCCATCCCCAACCTTCCAAATCAGTTCTCTTTTT 158
                   ||||||||||||||| |||||||||||||||| |||||||||||||||||||||||||||
Sbjct:    11756200 GGATTAGAAGCAATGCTGATGTTGGAGCCATCTCCAACCTTCCAAATCAGTTCTCTTTTT 11756259

Query:         159 CAACAACTTTGCGGCCCTCCAACAAGCTCCTCCATCTCCACGAAGGT 205
                   |||||||||||||||||||||||||||||||||||||||| ||||||
Sbjct:    11756260 CAACAACTTTGCGGCCCTCCAACAAGCTCCTCCATCTCCATGAAGGT 11756306
```

lncRNA3 against Aradu.A10: 107598427-107605282

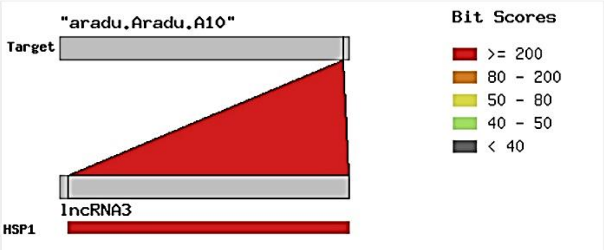

Alignment

HSP 1

Identity= 337/338 (99.7%) , Positive= 337/338 (99.7%) Query Matches 11 to 348 Hit Matches = 107604926 to 107605263

```
Query:         11 CCAGTTGTGTTGTCGAGAATTCGGCTTGAGATCACGAAGAGGGATTAGAAGATGTCTTTG 70
                  ||||||||||||||||||||||||||||||||||||||||||||||||||||||||||||
Sbjct:  107604926 CCAGTTGTGTTGTCGAGAATTCGGCTTGAGATCACGAAGAGGGATTAGAAGATGTCTTTG 107604985

Query:         71 CAATTGGATCTTATTGTCATGATGTCGATGTAGATATAGATTCTCGTTTTGTCTGAGTCA 130
                  ||||||||||||||||||||||||||||||||||||||||||||||||||||||||||||
Sbjct:  107604986 CAATTGGATCTTATTGTCATGATGTCGATGTAGATATAGATTCTCGTTTTGTCTGAGTCA 107605045

Query:        131 ATGATTTCGGATCAAGTCAAAAAGAATTATCTCCTGTCTAAAGAGGTGAAGTCTTTTTTG 190
                  ||||||||||||||||||||||||||||||||||||||||||||||||||||||||||||
Sbjct:  107605046 ATGATTTCGGATCAAGTCAAAAAGAATTATCTCCTGTCTAAAGAGGTGAAGTCTTTTTTG 107605105

Query:        191 ACCTAATTGAAGATCATCAACTCCGACAAAATGTAAGTCTATATCGACCTCAACTTCATG 250
                  ||||||||| ||||||||||||||||||||||||||||||||||||||||||||||||||
Sbjct:  107605106 ACCTAATTGGAGATCATCAACTCCGACAAAATGTAAGTCTATATCGACCTCAACTTCATG 107605165

Query:        251 AGAAGCAAGAAGGAAAGAAACAATTATAAGTGGTTAGAAACCTAAAATGTTTCCAAATTT 310
                  ||||||||||||||||||||||||||||||||||||||||||||||||||||||||||||
Sbjct:  107605166 AGAAGCAAGAAGGAAAGAAACAATTATAAGTGGTTAGAAACCTAAAATGTTTCCAAATTT 107605225

Query:        311 TGTAGTAAATGGAAAGGAATAGAGCAACCTGCATGACA 348
                  ||||||||||||||||||||||||||||||||||||||
Sbjct:  107605226 TGTAGTAAATGGAAAGGAATAGAGCAACCTGCATGACA 107605263
```

lncRNA4 against Aradu.A01: 1874505-1875866

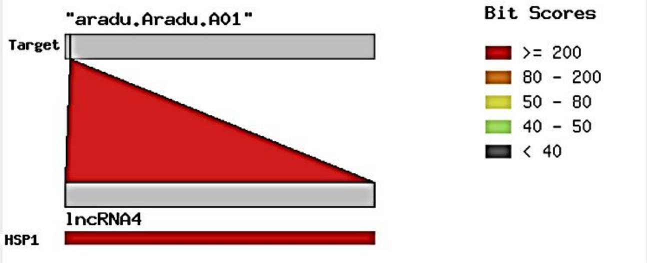

Alignment

HSP 1

Identity= 716/717 (99.86%) , Positive= 716/717 (99.86%) Query Matches 1 to 717 Hit Matches = 1875138 to 1875854

```
Query:          1 TCCCACATGCAAGCTGAGACATTAATTGAGCACCAAAGGTCTTAAACTCTTACTACCAGT 60
                  ||||||||||||||||||||||||||||||||||||||||||||||||||||||||||||
Sbjct:    1875138 TCCCACATGCAAGCTGAGACATTAATTGAGCACCAAAGGTCTTAAACTCTTACTACCAGT 1875197

Query:         61 TGTTGAATCTGAAGGCAAATGGCAAAGAAACCATTTACCATAGACATCAATACAAATGGC 120
                  ||||||||||||||||||||||||||||||||||||||||||||||||||||||||||||
Sbjct:    1875198 TGTTGAATCTGAAGGCAAATGGCAAAGAAACCATTTACCATAGACATCAATACAAATGGC 1875257

Query:        121 TAGAAAAAGCAAAGCTCCAAAGGTAAAAAAGGATCCATGCCTCATTAGAATATGAGCAAA 180
                  ||||||||||||||||||||||||||||||||||||||||||||||||||||||||||||
Sbjct:    1875258 TAGAAAAAGCAAAGCTCCAAAGGTAAAAAAGGATCCATGCCTCATTAGAATATGAGCAAA 1875317

Query:        181 GTGGGTAGTAGGAGGATCCCATTAGAAAAACCCTAATGAGAAAACCTCCATATTTGGTAA 240
                  ||||||||||||||||||||||||||||||||||||||||||||||||||||||||||||
Sbjct:    1875318 GTGGGTAGTAGGAGGATCCCATTAGAAAAACCCTAATGAGAAAACCTCCATATTTGGTAA 1875377

Query:        241 TTTGATGTTGCACAGCTATGACTCGAAACGCTTCCATTATTCACAGGGAAGGCGATCTTA 300
                  ||||||||||||||||||||||||||||||||||||||||||||||||||||||||||||
Sbjct:    1875378 TTTGATGTTGCACAGCTATGACTCGAAACGCTTCCATTATTCACAGGGAAGGCGATCTTA 1875437

Query:        301 AGGGCAGTGCTTAGAAATCAGAGAATAAGAACACTTTGCACAAAATTGGACATTCAAATA 360
                  ||||||||||||||||||||||||||||||||||||||||||||||||||||||||||||
Sbjct:    1875438 AGGGCAGTGCTTAGAAATCAGAGAATAAGAACACTTTGCACAAAATTGGACATTCAAATA 1875497

Query:        361 ACAGAAAATCAAACAAACTTTGGAGTATAGAATTTATCTCACAACGGATTCAAGAACAAT 420
                  ||||||||||||||||||||||||||||||||||||||||||||||||||||||||||||
Sbjct:    1875498 ACAGAAAATCAAACAAACTTTGGAGTATAGAATTTATCTCACAACGGATTCAAGAACAAT 1875557

Query:        421 GGATTGTAAAAACACATAATAAACGATTTGAGAATCCCTCAGATTATTTTTTTCCTCAAT 480
                  ||||||||||||||||||||||||||||||||||||||||||||||||||||||||||||
Sbjct:    1875558 GGATTGTAAAAACACATAATAAACGATTTGAGAATCCCTCAGATTATTTTTTTCCTCAAT 1875617

Query:        481 GGAATTCGGTCCACTACTTTAAATATTATAAATTGTCACAGGGAAGCATTAATATGTCAT 540
                  ||||||||||||||||||||||||||||||||| ||||||||||||||||||||||||||
Sbjct:    1875618 GGAATTCGGTCCACTACTTTAAATATTATAAATCGTCACAGGGAAGCATTAATATGTCAT 1875677

Query:        541 AAATCTGCATTAGAAATCAACCAAAACTCACCTAATCTTGAATACCAGCCAAAATAACAA 600
                  ||||||||||||||||||||||||||||||||||||||||||||||||||||||||||||
Sbjct:    1875678 AAATCTGCATTAGAAATCAACCAAAACTCACCTAATCTTGAATACCAGCCAAAATAACAA 1875737

Query:        601 TTTTTAAAAAATAATAATAATAAAGATATAAGAAAGAAATATTAATAAAAAGCATCCAAT 660
                  ||||||||||||||||||||||||||||||||||||||||||||||||||||||||||||
Sbjct:    1875738 TTTTTAAAAAATAATAATAATAAAGATATAAGAAAGAAATATTAATAAAAAGCATCCAAT 1875797

Query:        661 GAGATGAAAGAAATACTGAACAAACTCTCTTCTTAAGAAATCTCCGAAACCAGCCGC 717
                  |||||||||||||||||||||||||||||||||||||||||||||||||||||||||
Sbjct:    1875798 GAGATGAAAGAAATACTGAACAAACTCTCTTCTTAAGAAATCTCCGAAACCAGCCGC 1875854
```

lncRNA5 against Araip.B08: 42806631-42808445

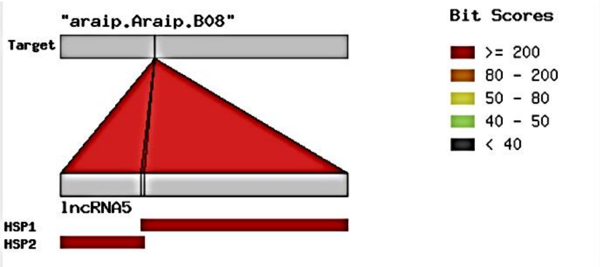

Alignment

HSP 1

Identity= 694/695 (99.86%) , Positive= 694/695 (99.86%) Query Matches 274 to 968 Hit Matches = 42807350 to 42808044

```
Query:          274 CACACAGCAAAATCTCAAATATATTATTATTTCATGAAAAGTGGAGAAGGTAAATAGGGT 333
                    ||||||||||||||||||||||||||||||||||||||||||||||||||||||||||||
Sbjct:     42807350 CACACAGCAAAATCTCAAATATATTATTATTTCATGAAAAGTGGAGAAGGTAAATAGGGT 42807409

Query:          334 GTTGCTTCAAAGTAACGAATGTTGTGGTAGTTTCACAATCAGAATTGGATTGGCTTGATG 393
                    ||||||||||||||||||||||||||||||||||||||||||||||||||||||||||||
Sbjct:     42807410 GTTGCTTCAAAGTAACGAATGTTGTGGTAGTTTCACAATCAGAATTGGATTGGCTTGATG 42807469

Query:          394 TTGTTGATAAGCCTAGAGGCAACAGCAATAGTGGGATAACAAATGAAATATTATAAAATT 453
                    ||||||||||||||||||||||||||||||||||||||||||||||||||||||||||||
Sbjct:     42807470 TTGTTGATAAGCCTAGAGGCAACAGCAATAGTGGGATAACAAATGAAATATTATAAAATT 42807529

Query:          454 GAAACCTCATTGCAATCATGCATGGTTGTCACTTGTCATTGCAACATTGACGTGGTGGTG 513
                    ||||||||||||||||||||||||||||||||||||||||||||||||||||||||||||
Sbjct:     42807530 GAAACCTCATTGCAATCATGCATGGTTGTCACTTGTCATTGCAACATTGACGTGGTGGTG 42807589

Query:          514 GCTGAGCTAATACAGCTCCTCGGGAACTGAAGGACCTACATTTATCTTAGTGGTATGAAT 573
                    ||||||||||||||||||||||||||||||||||||||||||||||||||||||||||||
Sbjct:     42807590 GCTGAGCTAATACAGCTCCTCGGGAACTGAAGGACCTACATTTATCTTAGTGGTATGAAT 42807649

Query:          574 GCATACACTTGATTACCCCTTCATCATCATTCATCACACCCCTCAACTCGTGAAAATAAA 633
                    ||||||||||||||||||||||||||||||||||||||||||||||||||||||||||||
Sbjct:     42807650 GCATACACTTGATTACCCCTTCATCATCATTCATCACACCCCTCAACTCGTGAAAATAAA 42807709

Query:          634 GTTACAAAATCCTAAATTGCTCCATATTTATTTATTTTAATTTCTCATTTTTCTTTATTA 693
                    ||||||||||||||||||||||||||||||||||||||||||||||||||||||||||||
Sbjct:     42807710 GTTACAAAATCCTAAATTGCTCCATATTTATTTATTTTAATTTCTCATTTTTCTTTATTA 42807769

Query:          694 GATTCTTAGCTTTTAAATTTACAAACTGTGAGATAGATCAATTTTTGTTTCTAACCTGTA 753
                    ||||||||||||||||||||||||||||||||||||||||||||||||||||||||||||
Sbjct:     42807770 GATTCTTAGCTTTTAAATTTACAAACTGTGAGATAGATCAATTTTTGTTTCTAACCTGTA 42807829

Query:          754 AAAGTATGAAATAATAAGGACTAACATATGCTCTTTGTAAATTTAGAAAATAATCTAGAT 813
                    ||||||||||||||||||||||||||||||||||||||||||||||||||||||||||||
Sbjct:     42807830 AAAGTATGAAATAATAAGGACTAACATATGCTCTTTGTAAATTTAGAAAATAATCTAGAT 42807889

Query:          814 CATTTATAAATTTTGGAACTAATTAAGGGATAAATAAAGACTAAAAACTGAATTGTTCAC 873
                    ||||||||||||||||||||||||||||||||||||||||||||||||||||||||||||
Sbjct:     42807890 CATTTATAAATTTTGGAACTAATTAAGGGATAAATAAAGACTAAAAACTGAATTGTTCAC 42807949

Query:          874 TCAACAAAAAAAAAAAAAGTCTGAAGACCAATTTGAAATTTTTCTACAAGAGTTATTCTC 933
                    ||||||||||||||||||||||||||||||||||||||||||||||||||||||||||||
Sbjct:     42807950 TCAACAAAAAAAAAAAAAGTCTGAAGACCAATTTGAAATTTTTCTACAAGAGTTATTCTC 42808009

Query:          934 AATTTATAAATTTAAGTACCGATAAATTCAACTGC 968
                    |||||||||||||||||||||| ||||||||||||
Sbjct:     42808010 AATTTATAAATTTAAGTACCGAAAAATTCAACTGC 42808044
```

HSP 2

Identity= 283/284 (99.65%) , Positive= 283/284 (99.65%) Query Matches 1 to 284 Hit Matches = 42806712 to 42806995

```
Query:            1 TCATCACCTCTCCAACTGACCTCTATTCTACTCTTAATCTTTCACCTCATTGTTGCACTT 60
                    ||||||||||||||||||||||||||||||||||||||||||||||||||||||||||||
Sbjct:     42806712 TCATCACCTCTCCAACTGACCTCTATTCTACTCTTAATCTTTCACCTCATTGTTGCACTT 42806771

Query:           61 ACTCCCACTAAATCAAATAAAATACTGAGAGTTAATTTAATTTTTGATTTTTCATTTTTG 120
                    ||||||||||||||||||||||||||||||||||||||||||||||||||||||||||||
Sbjct:     42806772 ACTCCCACTAAATCAAATAAAATACTGAGAGTTAATTTAATTTTTGATTTTTCATTTTTG 42806831

Query:          121 TCTCTTAATAATTTCTGCTTCTCAGTTTCTATTTTTTTTAAAATATTATAAAATGCAAAA 180
                    |||||||||||||||||||||||||||||||||||||||||||||||||||||| |||||
Sbjct:     42806832 TCTCTTAATAATTTCTGCTTCTCAGTTTCTATTTTTTTTAAAATATTATAAAATACAAAA 42806891

Query:          181 TAACTAAATCCTAATAATTATTAAAATTTAGCTAATCTGTGTCTTTTTTATGTAAATTTT 240
                    ||||||||||||||||||||||||||||||||||||||||||||||||||||||||||||
Sbjct:     42806892 TAACTAAATCCTAATAATTATTAAAATTTAGCTAATCTGTGTCTTTTTTATGTAAATTTT 42806951

Query:          241 CGTAATTAACAACATTAATATGTATTCTGAAGGCACACAGCAAA 284
                    ||||||||||||||||||||||||||||||||||||||||||||
Sbjct:     42806952 CGTAATTAACAACATTAATATGTATTCTGAAGGCACACAGCAAA 42806995
```

lncRNA6 against Araip.B06: 125052207-125052834

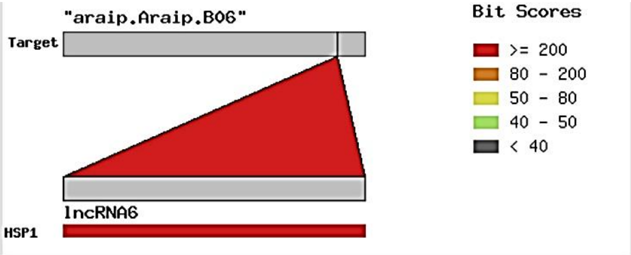

Alignment

HSP 1

Identity= 416/417 (99.76%) , Positive= 416/417 (99.76%) Query Matches 1 to 417 Hit Matches = 125052246 to 125052662

```
Query:          1 ATTCTCAACAATGATAGTAGTACATCATAAGTTAATTAATCTAAGTTTCAAAAAATTCCA 60
                  ||||||||||||||||||||||||||||||||||||||||||||||||||||||||||||
Sbjct:  125052246 ATTCTCAACAATGATAGTAGTACATCATAAGTTAATTAATCTAAGTTTCAAAAAATTCCA 125052305

Query:         61 GAACATGGCATCAATTTTGAAGAAAAGATTCCATTTCACTCACCTTGAGATGAACAAAGA 120
                  ||||||||||||||||||||||||||||||||||||||||||||||||||||||||||||
Sbjct:  125052306 GAACATGGCATCAATTTTGAAGAAAAGATTCCATTTCACTCACCTTGAGATGAACAAAGA 125052365

Query:        121 TGCATCCACACTGCTGCTTTTTTTTTTTCCCCATTTGATTCACACATTACAGATTCACTT 180
                  ||||||||||||||||||||||||||||||||||||||||||||||||||||||||||||
Sbjct:  125052366 TGCATCCACACTGCTGCTTTTTTTTTTTCCCCATTTGATTCACACATTACAGATTCACTT 125052425

Query:        181 TGTTCTCCTTCAGTTCAATCAAAACTACACATCAGCTTCATCATTTTGATTTAAAATGTC 240
                  ||||||||||||||||||||||||||||||||||||||||||||||||||| ||||||||
Sbjct:  125052426 TGTTCTCCTTCAGTTCAATCAAAACTACACATCAGCTTCATCATTTTGATTCAAAATGTC 125052485

Query:        241 AGCAACGGATAATCTTCTTTGATCCAAATTATTAGCACTTTCAACGTTGGGCCATTTTAC 300
                  ||||||||||||||||||||||||||||||||||||||||||||||||||||||||||||
Sbjct:  125052486 AGCAACGGATAATCTTCTTTGATCCAAATTATTAGCACTTTCAACGTTGGGCCATTTTAC 125052545

Query:        301 CTGGACTTGACTACCAAAATCAACCACAACTTGCTGATGCTGTGAACTCTGAACAATTAT 360
                  ||||||||||||||||||||||||||||||||||||||||||||||||||||||||||||
Sbjct:  125052546 CTGGACTTGACTACCAAAATCAACCACAACTTGCTGATGCTGTGAACTCTGAACAATTAT 125052605

Query:        361 TGCAACAACATCATCACTCCTCTGCTGATATTCCAAGGAATTGACACTGACAGTTGC 417
                  |||||||||||||||||||||||||||||||||||||||||||||||||||||||||
Sbjct:  125052606 TGCAACAACATCATCACTCCTCTGCTGATATTCCAAGGAATTGACACTGACAGTTGC 125052662
```

lncRNA7 against Araip.B10: 9092728-9099715

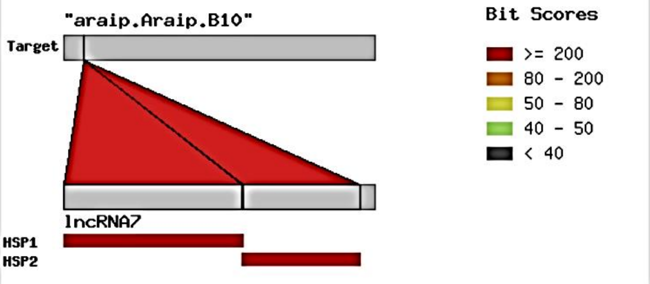

The image above shows the relationship between query and target for this particular BLAST hit.

Alignment

HSP 1

Identity= 515/520 (99.04%) , Positive= 515/520 (99.04%) Query Matches 1 to 518 Hit Matches = 9092895 to 9093414

```
Query:          1 AACTACGAG-AACCGAAACCATACCATGGTCGATTCCTCCCCAATGTCAAAGCA-CTTAC 58
                  ||||||||| |||||||||||||||||||||||||||||||||||||||||||| || ||
Sbjct:    9092895 AACTACGAGAAACCGAAACCATACCATGGTCGATTCCTCCCCAATGTCAAAGCACCTAAC 9092954

Query:         59 ATCAAGCTTCCAGGACTCCAAATCAATTCCAACAAGCTCTAATTTGCCAATTCAACACCG 118
                  |||||||||||| |||||||||||||||||||||||||||||||||||||||||||||||
Sbjct:    9092955 ATCAAGCTTCCAAGACTCCAAATCAATTCCAACAAGCTCTAATTTGCCAATTCAACACCG 9093014

Query:        119 AATTCAACACAATTCAATCTATTTTCATGAAATTCACCAAATTAACTAGGGTTCACATAA 178
                  ||||||||||||||||||||||||||||||||||||||||||||||||||||||||||||
Sbjct:    9093015 AATTCAACACAATTCAATCTATTTTCATGAAATTCACCAAATTAACTAGGGTTCACATAA 9093074

Query:        179 TTGAATAACCGCTAGGGTTTAGAGTTTCCTTACCTTACCCGCAGATTAATTAGACAAACC 238
                  |||||||||||| |||||||||||||||||||||||||||||||||||||||||||||||
Sbjct:    9093075 TTGAATAACCGCAAGGGTTTAGAGTTTCCTTACCTTACCCGCAGATTAATTAGACAAACC 9093134

Query:        239 CTTATACTTATCCACCACTAGAGTACCCTTAAACCATCAAAATTACAAAATCTCTCAATA 298
                  ||||||||||||||||||||||||||||||||||||||||||||||||||||||||||||
Sbjct:    9093135 CTTATACTTATCCACCACTAGAGTACCCTTAAACCATCAAAATTACAAAATCTCTCAATA 9093194

Query:        299 CCAAGAATTCAAATCACGAAACTGAAAAGGAAAGAAATGGACACAAAATCAGAATTTCTT 358
                  ||||||||||||||||||||||||||||||||||||||||||||||||||||||||||||
Sbjct:    9093195 CCAAGAATTCAAATCACGAAACTGAAAAGGAAAGAAATGGACACAAAATCAGAATTTCTT 9093254

Query:        359 TACCAAATTGTTGAGTGTGTTTTATAGAGAATTCCGAGACGAACACGTGACTGTTGACGG 418
                  ||||||||||||||||||||||||||||||||||||||||||||||||||||||||||||
Sbjct:    9093255 TACCAAATTGTTGAGTGTGTTTTATAGAGAATTCCGAGACGAACACGTGACTGTTGACGG 9093314

Query:        419 CTCCTCAATTGGAGGGCTATAAGGATGATTTGGACTTGGAAACTGGGTGATCGTTAGATT 478
                  ||||||||||||||||||||||||||||||||||||||||||||||||||||||||||||
Sbjct:    9093315 CTCCTCAATTGGAGGGCTATAAGGATGATTTGGACTTGGAAACTGGGTGATCGTTAGATT 9093374

Query:        479 GTTGAGGCTCCAAATACTAGATCCAGAACAATATATATCC 518
                  ||||||||||||||||||||||||||||||||||||||||
Sbjct:    9093375 GTTGAGGCTCCAAATACTAGATCCAGAACAATATATATCC 9093414
```

HSP 2

Identity= 341/342 (99.71%) , Positive= 341/342 (99.71%) Query Matches 517 to 858 Hit Matches = 9098042 to 9098383

```
Query:        517 CCCATAATTCTTAGCGAAGACTGAAATCACACTTGCTGAAAGCTGTGCTTTGATCACCAA 576
                  ||||||||||||||||||||||||||||||||||||||||||||||||||||||||||||
Sbjct:    9098042 CCCATAATTCTTAGCGAAGACTGAAATCACACTTGCTGAAAGCTGTGCTTTGATCACCAA 9098101

Query:        577 CCTTAAAACACAAATGCCAAGAAGTAACATGTTGCAGTAGTAGTGATAAAGCTATACCAA 636
                  ||||||||||||||||||||||||||||||||||||||||||||||||||||||||||||
Sbjct:    9098102 CCTTAAAACACAAATGCCAAGAAGTAACATGTTGCAGTAGTAGTGATAAAGCTATACCAA 9098161

Query:        637 ATATGATTAAAACAGTGGTTAGCCCAACATATATAAACAAGTTCAACAAATGATGCACAA 696
                  ||||||||||||||||||||||||||||||||||||||||||||||||||||||||||||
Sbjct:    9098162 ATATGATTAAAACAGTGGTTAGCCCAACATATATAAACAAGTTCAACAAATGATGCACAA 9098221

Query:        697 TAATATTTCCATTTTGCAAAGCCCTAATTATAAATCAGTAAATTCACATCGCAACTACAG 756
                  ||||||||||||||||||||||||||||||||||||||||||||||||||||||||||||
Sbjct:    9098222 TAATATTTCCATTTTGCAAAGCCCTAATTATAAATCAGTAAATTCACATCGCAACTACAG 9098281

Query:        757 CATTTTAAAAATATAAACGGATCCTTTGGCAATCAATAATACCAAAAATACTTTTTGCGT 816
                  |||||||||||||||||| |||||||||||||||||||||||||||||||||||||||||
Sbjct:    9098282 CATTTTAAAAATATAAACAGATCCTTTGGCAATCAATAATACCAAAAATACTTTTTGCGT 9098341

Query:        817 TGTTTGCCTAATGGCACAAGAGTAGTAGATTCCACTTCCACC 858
                  ||||||||||||||||||||||||||||||||||||||||||
Sbjct:    9098342 TGTTTGCCTAATGGCACAAGAGTAGTAGATTCCACTTCCACC 9098383
```
