# Supplementary material for: Discovery, identification, and functional characterization of long noncoding RNAs in Arachis hypogaea L
Source: BMC Plant Biol. 2020 Jul 2;20:308. doi: 10.1186/s12870-020-02510-4 (PMC7330965; doi:10.1186/s12870-020-02510-4)
Supplement: Supplementary file 12 — Additional file 12 Figure S2. Sanger sequencing of five lncRNAs (T1–5). [file 12870_2020_2510_MOESM12_ESM.pdf]

>T1
CAGTTCTTTATAACCTCATGGGTAATACTCATGTTAAACTAAGGTAACAATG
AATATTTATAATCCAAAAAGTAAAAAATAATTTAACAAAGGAACAGCTGCC
AGCCGGAACATTTATTGCACTGAGAGTTAACATTGAGACCCTTTGAAAATC
TGAGTGAACTCTATGTCCTCCTAAGTCCTCACCAGAGGCTCCTGCTGTTACT
CGTATAATATCTTCAATAAGTGCAAAGTTCCCCAATATATCATGAGCACCACT
CTGGGTTCCCCTTCCTTCGAGAAGATTAGCCGGAGCAGCGTCTTCATACTC
CCTTATGAACGTTTAGATGCCTGAAGGATTAAAGTGGATTTTTTCCCGCCAA
CCTCTTGCACAAATGAAACCAGCGCTTAAAAGAGGATCGGTTTCATCCCCG
TTGGCACTATAAACTCCATCCTTTAAAAAAGGGTCCTCATCTCCACCAATTG
CTGATGTGTCAATCTGTCGAAGCCATTGGATCACCTCACAATTGCCATCATG
AAAGCGAAAAGACAGCTCCATGACCACAAATCTAGCTTTTCAATTTGACCC
ATATTCCAATCAGCAATCACTCCTACAAATCATCCCCCTCCCTAAACCCTAGT
AGTG

>T2
CAGTTCTTTATAACCTCATGGGTAATACTCATGTTAAACTAAGGTAACAATG
AATATTTATAATCCAAAAAGTAAAAAATAATTTAACAAAGGAACAGCTGCC
AGCCGGAACATTTATTGCACTGAGAGTTAACATTGAGACCCTTTGAAAATC
TGAGTGAACTCTATGTCCTCCTAAGTCCTCACCAGAGGCTCCTGCTGTTACT
CGTATAATATCTTCAATAAGTGCAAAGTTCCCCAATATATCATGAGCACCACT
CTGGGTTCCCCTTCCTTCGAGAAGATTAGCCGGAGCAGCGTCTTCATACTC
CCTTATGAACGTTTAGATGCCTGAAGGATTAAAGTGGATTTTTTCCCGCCAA
CCTCTTGCACAAATGAAAACCAGCGCTTAAAAGAGGATCGGTTTCATCCCC
GTTGGCACTATAAACTCCATCCTTTAAAAAAGGGTCCTCATCTCCACCAATT
GCTGATGTGTCAATCTGAAACGGAATGTAGCATTTCGATTGAGAGGTTGAC
TTATAAACATAGGCTCTTTACATTAGAATTCCGACCCCATACATAGAGTAAAT
CTCCATATTTGGAGCATGTGGTAATCTGTCGAAGCCATTGGATCACCTCACA
ATTGCCATCATGAAAGCGAAAAGACAGCTCCATGACCACAAATCTAGCTTT
TCAATTTGACCCATATTCCAATCAGCAATCACTCCTACAAATCATCCCCCTC
CCTAAACCCTAGTAGTG

>T3
TGCACAAATGAAACCAGCGCTTAAAAGAGGATCGGTTTCATCCCCGTTGGC
ACTATAAACTCCATCCTTTAAAAAAAGGGTCCTCATCTCCACCAATTGCTGA
TGTGTCAATCTGAAACGGAATGTAGCATTCCGATTGAGAGGTTGACTTATAA
ACATAGGCTCTTTACATTAGAATTCCGACCCCATACATAGAGTAAATCTCCAT
ATTTGGAGCATGTGGTAATCTAGTAGTGCCACCGCCGCCACTGCAAACACC
AGAGCGGACCATCAAAAGCAAATCATCATCCATCATGCCCATCTCTCATAAA
AGAGGGAAACAAAATATGCATTGATACAATAAGTACCCAAGAAAATAAAGA
CCAAAAGATCAAAGAAACAATAAAGAATAAAATAAAGGAACCAGCGGTTG
AAATTACACCATACAGATTTTAAAGAATTTCC

>T4

TGCACAAATGAAACCAGCGCTTAAAAGAGGATCGGTTTCATCCCCGTTGGC
ACTATAAACTCCATCCTTTAAAAAAGGGTCCTCATCTCCACCAATTGCTGAT
GTGTCAATCTGAAACGGAATGTAGCATTTCGATTGAGAGGTTGACTTATAA
ACATAGGCTCTTTACATTAGAATTCCGACCCCATACATAGAGTAAATCTCCAT
ATTTGGAGCATGTGGTAATCTGTCGAAGCCATTGGATCACCTCACAATTGCC
ATCATGAAAGCGAAAAGACAGCTCCATGACCACAAATCTAGCTTTTCAATT
TGACCCATATTCCAATCAGCAATCACTCCTACAAATCATCCCCCTCCCTAAA
CCCTACCCTTAACTTTCTTGAAAATTTTCTGACCTCATTTTCATATGCAGTAA
AACTGTATTGCAATTTGAGAACCGAAACCAACAACAACCTCAATCGCTCTC
TTATATATCTAGTAGTGCCACCGCCGCCACTGCAAACACCAGAGCGGACCA
TCAAAAGCAAATCATCATCCATCATGCCCATCTCTCATAAAAGAGGGAAAC
AAAATATGCATTGATACAATAAGTACCCAAGAAAATAAAGACCAAAAGATC
AAAGAAACAATAAAGAATAAAATAAAGGAACCAGCGGTTGAAATTACACC
ATACAGATTTTAAAGAATTTCC

>T5
CCTCATCTCCACCAATTGCTGATGTGTCAATCTGAAACGGAATGTAGCATTC
CGATTGAGAGGTTGACTTATAAACATAGGCTCTTTACATTAGAATTCCGACC
CCATACATAGAGTAAATCTCCATATTTGGAGCATGTGGTAATCTGTCGAAGC
CATTGGATCACCTCACAATTGCCATCATGAAAGCGAAAAGACAGCTCCATG
ACCACAAATCTAGCTTTTCAATTTGACCCATATTCCAATCAGCAATCTCCCA
CAAATCATCCCCCTCCCTAAACCCTACCCTAGTAGTGCCACCGCCGCCACT
GCAAACACCAGAGCGGACCATCAAAAGCAAATCATCATCCATCATGCCCAT
CTCTCATAAAAGAGGGAAACAAAATATGCATTGATACAATAAGTACCCAAG
AAAATAAAGACCAAAAGATCAAAGAAACAATAAAGAATAAAATAAAGGAA
CCCAGCGGTTGAAATTACACCATACAG

Note: the red words in T1and T2 indicate the primers of 911-F and 911-R; the blue words in T3 and T4 show the primers of 806-F and 806-R; and the green words in T5 show the primers of 269-F and 269-R.
