# Supplementary figures and images for: Discovery, identification, and functional characterization of long noncoding RNAs in Arachis hypogaea L
Source: BMC Plant Biol. 2020 Jul 2;20:308. doi: 10.1186/s12870-020-02510-4 (PMC7330965; doi:10.1186/s12870-020-02510-4)

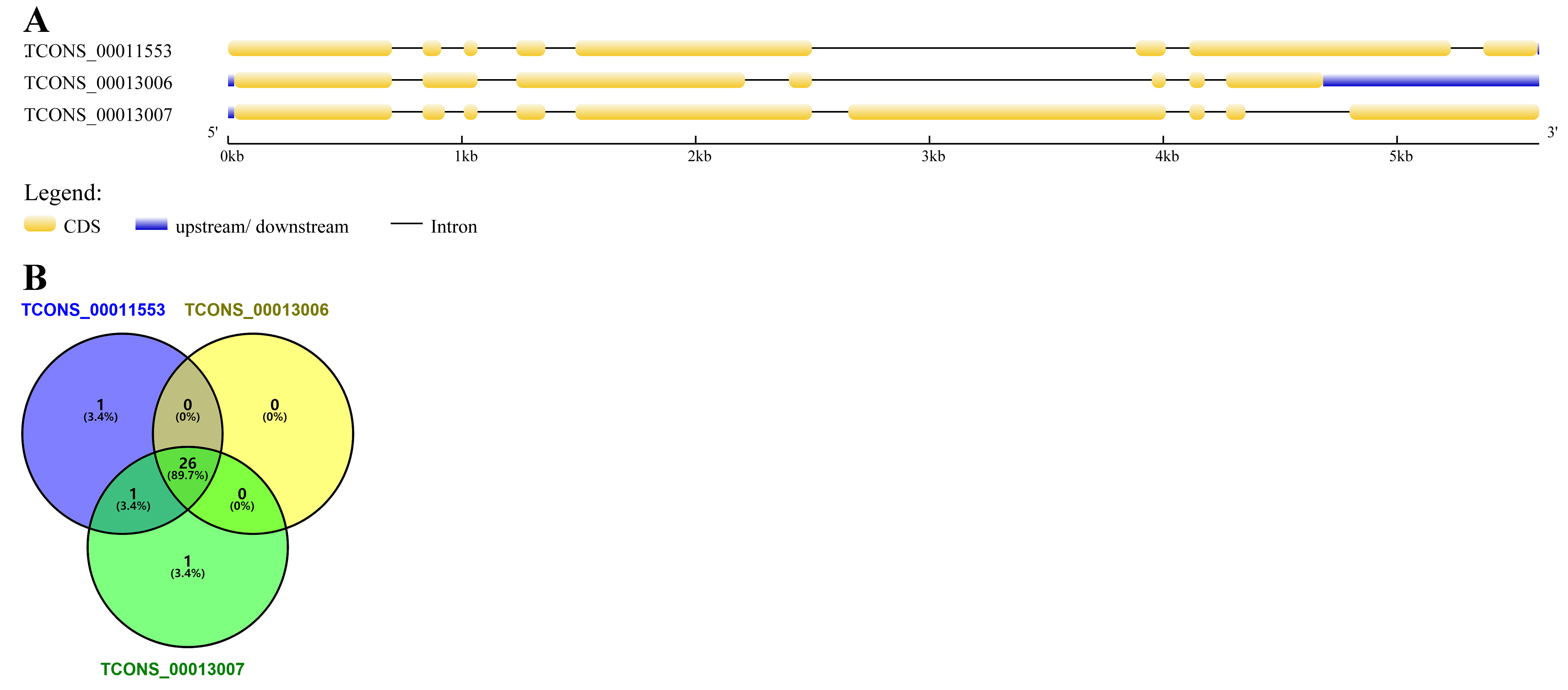

Supplement: Supplementary file 13 — Additional file 13: Figure S3. AS Events of LncRNAs and different AS isoforms had different target genes. [file 12870_2020_2510_MOESM13_ESM.jpg]

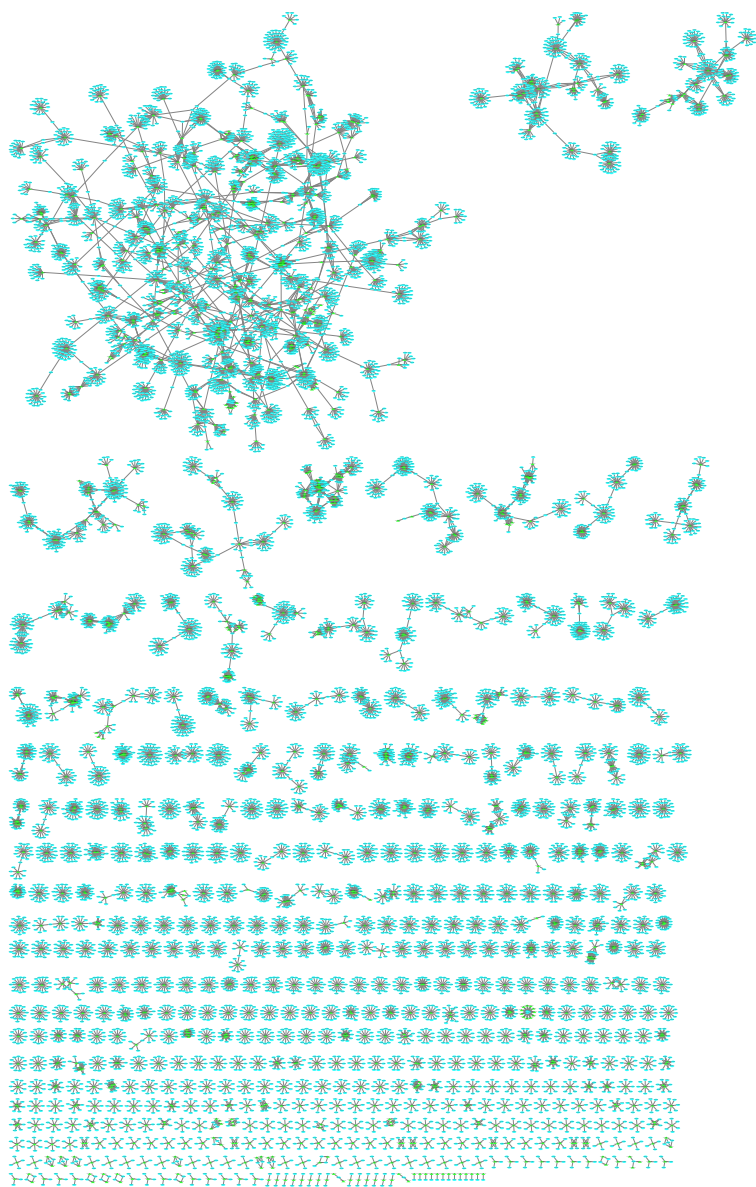

Supplement: Supplementary file 15 — Additional file 15: Figure S5. The lncRNAs-protein interaction networks. [file 12870_2020_2510_MOESM15_ESM.pdf]
