# Supplementary material for: Discovery, identification, and functional characterization of long noncoding RNAs in Arachis hypogaea L
Source: BMC Plant Biol. 2020 Jul 2;20:308. doi: 10.1186/s12870-020-02510-4 (PMC7330965; doi:10.1186/s12870-020-02510-4)
Supplement: Supplementary file 14 — Additional file 14: Figure S4. Sanger sequencing of five lncRNAs (L1.1–1.5). [file 12870_2020_2510_MOESM14_ESM.pdf]

>L1.1
ATGGCGGAGAAAGTATTCAGCGCGAAAGAGATCTTCGCCGCCGATTCGTCC
AACATCTTCATAATGATTCTGGCATTGTTGATTTGGCTTGGAGCTATACACCT
CAATTTCCTCTTTATTCTCTTGTTGATATTCCTTCCTCTCTCCAAATCACTCTT
GCTTTTTGGTTTGCTGGTTGTGTTGGTGGTGATTCCGGTGGACGATAAGAG
CAGATTGGGAAGAAGGTTGGCGAGGTCCTTAAGCAAGTATGCTTGTAGTTA
CTTCCCGGTCACGCTTCATGTAGAGGATATGAATGCATTTGATCCTAATCGC
GCTTATGTTTTTGGTTATGAACCACACTCGGTTTTGCCAATTGGTGTCATTG
CGCTAGCTGAAAGCATTGGTTGGATGCCACTTCCAAAATTGAAAGTTCTTG
CTAGCAGTGCAATTTTTTACATACCGGTGTTGAGACACTTATGGACATGGTT
TGGCGTCACACGCGCAGCAAAGAAAAATTTTATCTCCCAGTTATCAGCTGG
ATACTCTTGCGTTTTAATACCTGGTGGAGTGCAAGAAACATTTTTTATGAAG
CATGGCTCCGAGATTGCTTTCCTTAAAACAAGAAGAGGATTTGTTCGTATAG
CAATGGAGCGTGGTCATCCCTTGGTTCCAACTTTTTGCTTTGGCCAGTCAG
ATGTCTACAAGTGGTGGAAACCAAGTGGGACGTTGGTTATGAAACTTTCAA
GAGCTATAAAGTTCGCCCCAATATTTTACTGGGGGATTTTCGGAACACCAAT
ACCGTTCAGACGTCCAATGTATATAGCAGTGGGCAGACCAATTGAACCTCC
CAAAAATCCAGAACCAACCCAGGAGGAGGTTGACAAAGTGCTTATTCAGT
TTGTGGATGCACTACAAGATCTATTCGAAAGACACAAAGCTCGGGCTGGAT
ATCCAAAGCTTGAGTTAAGAATCCTATGA

>L1.2
ATGGCGGAGAAAGTATTCAGCGCGAAAGGGATCTTCGCCGCCGGTTCGTCC
AACATCTTCATAATGATTCTGGCATTGTTGATTTGGCTCGGAGCTATACACCT
CAATTTCCTCTTTATTCTCTTGTTGATATTCCTTCCTCTCTCCAAATCACTCTT
GCTTTTTGGTTTGCTGGTTGTGTTGGTGGTGATTCCGGTGGACGATAAGAG
CAGATTGGGAAGAAGGTTGGCGAGGTCCTTAAGCAAGTATGCTTGTAGTTA
CTTCCCGGTCACGCTTCATGTAGAGGATATGAATGCGTTTGATCCTAATCGC
GCTTATGTGACAAGAGTATGCACAAAATGCAGTTTTTGGTTATGAACCACA
CTCGGTTTTGCCAATTGGTGTCATTGCACTAGCTGAAAGCATTGGTTGGATG
CCACTTCCAAAATTGAAAGTTCTTGCTAGCAGTGCAATTTTTTACATACCGG
TGTTGAGACACTTATGGACATGGTTTGGCGTCACACGCGCAGCAAAGAAA
AATTTTATCTCCCAGTTATCAGCTGGATACTCTTGCGTTTTAATACCTGGTGG
AGTGCAAGAAACATTTTTTATGAAGCATGGCTCCGAGATTGCTTTCCTTAAA
ACAAGAAGAGGATTTGTTCGTATAGCAATGGAGCGTGGTCATCCCTTGGTT
CCAACTTTTTGCTTTGGTCAGTCAGATGTCTACAAGTGGTGGAAACCAAGT
GGGACGTTGGTTATGAAACTTTCAAGAGCTATAAAGTTCGCCCCAATATTTT
ACTGGGGGATTTTCGGAACACCAATACCGTTCAGACGTCCAATGTATATAGC
AGTGGGCAGACCAATTGAACCTCCTAAAAATCCAGAACCAACCCAGGAGG
AGGTTGACAAAGTGCTTATTCAGTTTGTGGATGCACTACAAGATCTATTCGA
AAGACACAAAGCTCGGGCTGGATATCCAAAGCTTGAGTTAAGAATCCTATG
A

>L1.3

ATGGCGGAGAAAGTATTCAGCGCGAAAGAGATCTTCGCCGCCGATTCGTCC
AACATCTTCATAGTGATTCTGACATTGTTGATTTGGCTCGGAGCTATACACC
TCAATTTCCTCTTTATTCTCTTGTTGATATTCCTTCCTCTCTCCAAATCACTCT
TGCTTTTTGGTTTGCTGGTTGTGTTGGTGGTGATTCCGGTGGACGATAAGA
GCAGATTGGGAAGAAGGTTGGCGAGGTCCTTAAGCAAGTATGCTTGTAGTT
ACTTCCCGGTCACGCTTCATGTAGAGGATATGAATGCATTTGATCCTAATCG
CGCTTATGTTTTTGGTTATGAACCACACTCGGTTTTGCCAATTGGTGTCATT
GCACTAGCTGAAAGCATTGGTTGGATGCCACTTCCAAAATTGAAAGTTCTT
GCTAGCAGTGCAATTTTTTACATACCGGTGTTGAGACACTTATGGACATGGT
TTGGCGTCACACGCGCAGCAAAGAAAAATTTTATCTCCCAGTTATCAGCTG
GATACTCTTGCGTTTTAATACCTGGTGGAGTGCAAGAAACATTTTTTATGAA
GCATGGCTCCGAGATTGCTTTCCTTAAAACAAGAAGAGGATTTGTCCGTAT
AGCAATGGAGCGTGGCCATCCCTTGGTTCCAACTTTTTGCTTTGGTCAGAA
CACCAATACCGTTCAGACGTCCAATGTATATAGCAGTGGGCAGACCAATTG
AACCTCCCAAAAATCCAGAACCAACCCAGGAGGAGGTTGACAAAGTGCTT
ATTCAGTTTGTGGATGCACTACAAGATCTGTTCGAAAGACACAAAGCTCGG
GCTGGATATC CAAAGCTTGAGTTAAGAATCCTATGA

>L1.4
ATGGCGGAGAAAGTATTCAGCGCGAAAGAGATCTTCGCCGCCGATTCGTCC
AACATCTTCATAATGATTCTGGCATTGTTGATTTGGCTCGGAGCTATACACCT
CAATTTCCTCTTTATTCTCTTGTTGATATTCCTTCCTCTCTCCAAATCACTCTT
GCTTTTTGGTTTGCTGGTTGTGTTGGTGGTGATTCCGGTGGACGATAAGAG
CAGATTGGGAAGAAGGTTGGCGAGGTCCTTAAGCAAGTATGCTTGTAGTTA
CTTCCCGGTCACGCTTCATGTAGAGGATATGAATGCATTTGATCCTAATCGC
GCTTATGTTTTTGGTTATGAACCACACTCGGTTTTGCCAATTGGTGTCATTG
CACTAGCTGAAAGCATTGGTTGGATGCCACTTCCAAAATTGAAAGTTCTTG
CTAGCAGTGCAATTTTTTACATACCGGTGTTGAGACACTTATGGACATGGTT
TGGCGTCACACGCGCAGCAAAGAAAAATTTTATCTCCCAGTTATCAGCTGA
TACTCTTGCGTTTTAATACCTGGTGGAGTGCAAGAAACATTTTTTATGAAGC
ATGGCTCCGAGATTGCTTTCCTTAAAACAAGAAGAGGATTTGTTCGTATAGC
AATGGAGCGTGGTCATCCCTTGGTTCCAACTTTTTGCTTTGGTCAGGTACTT
TTGGCTTTATAAAACTATATATTACTCCAAAATTTATTTGTGTATAGTTTCCTT
ATCTGTTAATTTCACTCAATTTTCTTTGCTCATGCAATCAAGTTAACCCCCTT
TTGGTGTAACTTTGCCATAGTCAGATGTCTACAAGTGGTGGAAACCAAGTG
GGACGTTGGTTATGAAACTTTCAAGAGCTATAAAGTTCGCCCCAATATTTTA
CTGGGGGATTTTCGGGTGTGTGTTTCTTTTATGCATATATGTGAACTGTTTTC
ACTGGCCAAGGGTAATTCAACAACAGCTGAAGCTGAATACTATTTATGGTT
AAGAATAGCCTGTGTTAACAAACATTTTTTTCTATCAACATTGATGATTCTTG
TAACGAAACAGCATCGCATTGCCTGATTCACATAGGCAACTCCATATATGTA
TTGAAAATACTTATAAAAGCCTTGAATAATTGATTTGAGTAGAATAGCTACT
GCACTAGTATAGTTAAGCATGTAAGATGCTGTTGTATACTTGTATGGAGCAA
TTTCGGAAACTGATATTTGAAAGCCACTCCTTTTCAGAACACCAATACCGTT
CAGACGTCCAATGTATATAGCAGTGGGCAGACCAATTGAACCTCCCAAAAA

TCCAGAACCAACCCAGGAGGAGGTTGACAAAGTGCTTATTCAGTTTGTGG
ATGCACTACAAGATCTATTCGAAAGACACAAAGCTCGGGCTGGATATCCAA
AGCTTGAGTTAAGAATCCTATGA

>L1.5
ATGGCGGAGAAAGTATTCAGCGCGAAAGAGATCTTCGCCGCCGATTCGTCC
AACATCTTCATAATGATTCTGGCATTGTTGATTTGGCTCGGAGCTATACACCT
CAATTTCCTCTTTATTCTCTTGTTGATATTCCTTCCTCTCTCCAAATCACTCTT
GCTTTTTGGTTTGCTGGTTGTGTTGGTGGTGATTCCGGTGGACGATAAGAG
CAGATTGGGAAGAAGGTTGGCGAGGTCCTTAAGCAAGTATGCTTGTAGTTA
CTTCCCGGTCACGCTTCATGTAGAGGATATGAATGCATTTGATCCTAATCGC
GCTTATGTTTTTGGTTATGAACCACACTCGGTTTTGCCAATTGGTGTCATTG
CACTAGCTGAAAGCATTGGTTGGATGCCACTTCCAAAATTGAAAGTTCTTG
CTAGTAGTGCAATTTTTTACATACCGGTGTTGAGACACTTATGGACATGGTT
TGGCGTCACACGCGCAGCAAAGAAAAATTTTATCTCCCAGTTATCAGCTGG
ATACTCTTGCGTTTTAATACCTGGTGGAGAGCAAGAAACATTTTTTATGAAG
CATGGCTCCGAGATTGCTTTCCTTAAAACAAGAAGAGGATTTGTTCGTATAG
CAATGGAGCGTGGTCATCCCTTGGTTCCAACTTTTTGCTTTGAACACCAATA
CCGTTCAGACGTCCAATGTATATAGCAGTGGGCAGACCAATTGAACCTCCC
AAAAATCCAGAACCAACCCAGGAGGAGGTTGACAAAGTGCTTATTCAGTT
TGTGGATGCACTACAAGATCTATTCGAAAGACACAAAGCTCGGGCTGGATA
TCCAAAGCTTGAGTTAAGAATCCTATGA

Note: the red words indicate the position of the primers.
